# Theranostic applications of selenium nanomedicines against lung cancer

**DOI:** 10.1186/s12951-023-01825-2

**Published:** 2023-03-20

**Authors:** Shaowei Liu, Weifeng Wei, Jinlin Wang, Tianfeng Chen

**Affiliations:** 1 Pulmonary and Critical Care Medicine, Guangzhou Institute of Respiratory Health, National Clinical Research Center for Respiratory Disease, National Center for Respiratory Medicine, State Key Laboratory of Respiratory Diseases, The First Affiliated Hospital of Guangzhou Medical University, Guangzhou, 510120 China; 2 College of Chemistry and Materials Science, Guangdong Provincial Key Laboratory of Functional Supramolecular Coordination Materials and Applications, Jinan University, Guangzhou, 510632 China

**Keywords:** Lung cancer, Selenium (Se), Selenium nanomedicines, Nanotechnology, SeNPs, Theranostic applications

## Abstract

The incidence and mortality rates of lung cancer are among the highest in the world. Traditional treatment methods include surgery, chemotherapy, and radiotherapy. Although rapid progress has been achieved in the past decade, treatment limitations remain. It is therefore imperative to identify safer and more effective therapeutic methods, and research is currently being conducted to identify more efficient and less harmful drugs. In recent years, the discovery of antitumor drugs based on the essential trace element selenium (Se) has provided good prospects for lung cancer treatments. In particular, compared to inorganic Se (Inorg-Se) and organic Se (Org-Se), Se nanomedicine (Se nanoparticles; SeNPs) shows much higher bioavailability and antioxidant activity and lower toxicity. SeNPs can also be used as a drug delivery carrier to better regulate protein and DNA biosynthesis and protein kinase C activity, thus playing a role in inhibiting cancer cell proliferation. SeNPs can also effectively activate antigen-presenting cells to stimulate cell immunity, exert regulatory effects on innate and regulatory immunity, and enhance lung cancer immunotherapy. This review summarizes the application of Se-based species and materials in lung cancer diagnosis, including fluorescence, MR, CT, photoacoustic imaging and other diagnostic methods, as well as treatments, including direct killing, radiosensitization, chemotherapeutic sensitization, photothermodynamics, and enhanced immunotherapy. In addition, the application prospects and challenges of Se-based drugs in lung cancer are examined, as well as their forecasted future clinical applications and sustainable development.

**Graphical Abstract:**

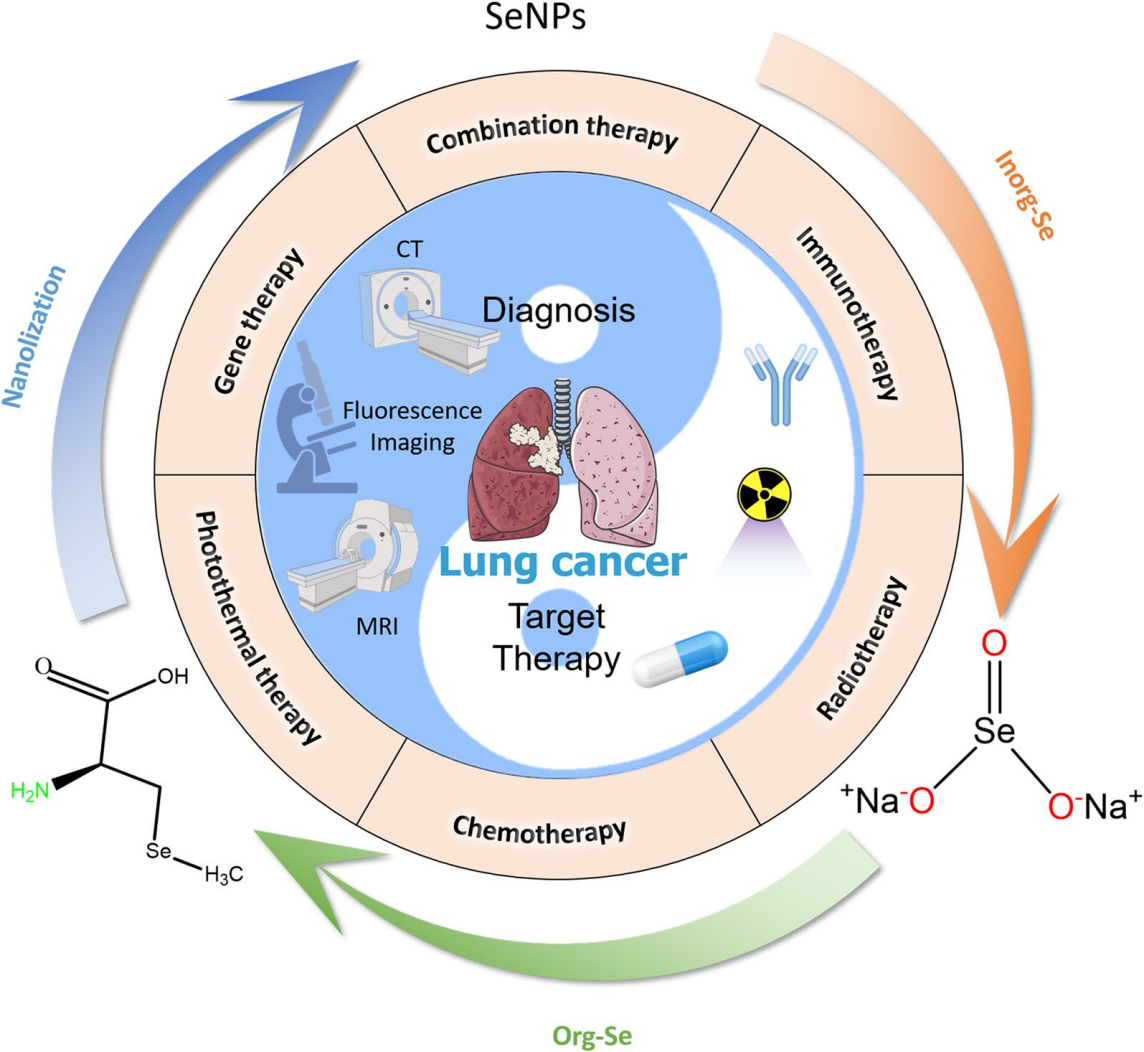

## Introduction

According to 2020 global cancer statistics, the cancer with the highest incidence (11.4%) and mortality (18%) worldwide is lung cancer [1]. The 5-year relative survival rate for lung cancer is 19%, and that of patients with small-cell lung cancer (SCLC) is only 2% [2]. The aggressive progression of lung cancer and the high mortality rate of cancer patients have aroused great concern among scientists [3]. The major forms of therapy for lung malignancies continue to be surgery, chemotherapy, and radiotherapy (RT) [4]. However, surgery is a highly traumatic local therapy option. Chemotherapy and radiation cause side effects, such nausea, vomiting, and bone marrow suppression, thus, it difficult to employ these treatments in clinical settings. Recently, even though immunotherapies and gene mutation therapies that specifically targeted lung cancer have demonstrated excellent therapeutic effects, acquired resistance to epidermal growth factor receptor (EGFR)-tyrosine kinase inhibitors (TKIs) is unavoidable. Most patients lose their ability to respond to TKIs after 1 year, their condition worsens quickly [5], and there is only a 20% overall response rate for immunotherapy [6]. Therefore, safer and more efficient treatment modalities are urgently needed in the therapeutic setting.

Drug research and development to diagnose and treat advanced lung cancer has advanced quickly in recent years. Examples include ALK inhibitors (crizotinib [7], ceritinib [8], lorlatinib [9]), second-generation EGFR-TKIs (afatinib [10], dacomitinib [11]), and third-generation EGFR-TKIs (osimertinib [12], olmutinib [13]). In particular, immunotherapy (nivolumab [14], pembrolizumab [15]) has been used widely and achieved remarkable results. By focusing on PD-1 on the surface of T cells, these immunotherapies aim to enhance the body's immune system and the tumor immune microenvironment (TIME). TIME consists of environmental chemicals, immune cells, and cytokines [16]. Recent research has demonstrated that peripheral chemicals contribute significantly to the tumor microenvironment. According to a recent study, cancers are not sterile environments as previously thought since they contain microorganisms that help tumor cells spread by extending their ability to survive after leaving the initial tumor [17]. TIME also involves nutritional regulation. According to studies, patients with malignant pleural effusion caused by advanced lung cancer show serum levels of selenium (Se) that are lower than those of healthy individuals [18]. This further demonstrates the significance of Se.

Se, which is known as selenoprotein, is a unique and necessary trace element [19, 20] and plays a significant role in many physiological processes in humans. Se has been studied for many years as a cancer treatment, and various studies have examined its function as a chemopreventive agent in lung cancer [21]. According to the findings of epidemiological and clinical studies, Se supplementation can considerably lower the incidence of lung cancer in people with low baseline levels of Se. Additional research verified the crucial role of Se in preventing oxidative DNA damage and increasing DNA repair, indicating its potential significant role in carcinogenesis [22, 23]. Although the importance of Se is becoming more widely acknowledged, the use of existing Se supplements (inorganic selenium [Inorg-Se] and organic selenium [Org-Se]) is constrained by issues such as poor absorption and increased toxicity*.* Se supplementation, at a serum level of 106 ng/mL, decreased the risk of lung cancer, but a higher Se level (121.6 ng/mL) increased the risk of lung cancer and was associated with diabetes [24]. SeMet exposure has also been shown to enhance radiosensitivity in human lung cancer cell lines without damaging normal lung cell lines. The doses used to treat cells in a study may be much higher than those needed for research in vivo [25]. Therefore, the appropriate dose for conducting in vivo studies must be determined. Creating novel systems as Se compound transporters is critical. The most recent advancements in nanotechnology have addressed this urgent problem.

Nanotechnology is a high-tech discipline that combines basic multidisciplinary research with basic applications to create materials or structures that range in size from 0.1 nm to 100 nm. Currently, nanotechnology is being used in medicine, creating the field of nanomedicine. Nanomedicine is a branch of science and technology in which molecular instruments and knowledge of the human body at the molecular level are used to identify, treat, and prevent disease and traumatic injury while reducing pain and preserving and enhancing human health [26]. As a result, the diagnosis and treatment of cancers is the most popular area of study in nanomedicine. A nanoscale drug delivery system is a device for concentrating medications and therapeutic substances in specific tissues and organs [27, 28]. Strong cell uptake, low toxicity to normal cells, and increased anticancer activity are all benefits of using nanoparticle-mediated drug delivery in cancer therapy [29–31]. Consequently, nanoscale drug delivery systems have emerged as a popular area of study. Similar to recent hot research topics, nanodrugs are increasingly becoming apparent. When treating advanced ovarian cancer, for instance, doxorubicin (DOX) hydrochloride liposome nanomedicine (Doxil) is frequently employed [32, 33]. Advanced ovarian cancer, breast cancer, and lung cancer are treated regularly with nanoparticle albumin-bound paclitaxel (Abraxane^®^) [34–37]. Se possesses antitumor properties as well, and SeNPs are utilized more often and are less hazardous. SeNPs that have undergone nanotechnology modifications have proven to be less harmful and more popular. To enhance immunotherapy against prostate cancer, Lai et al. [38] developed a selenium-containing ruthenium complex with natural killer cells. Liao et al. [39] investigated the use of selenium nanoparticles (SeNPs) to treat tumors by upregulating mir-16.

**Fig. 1:**
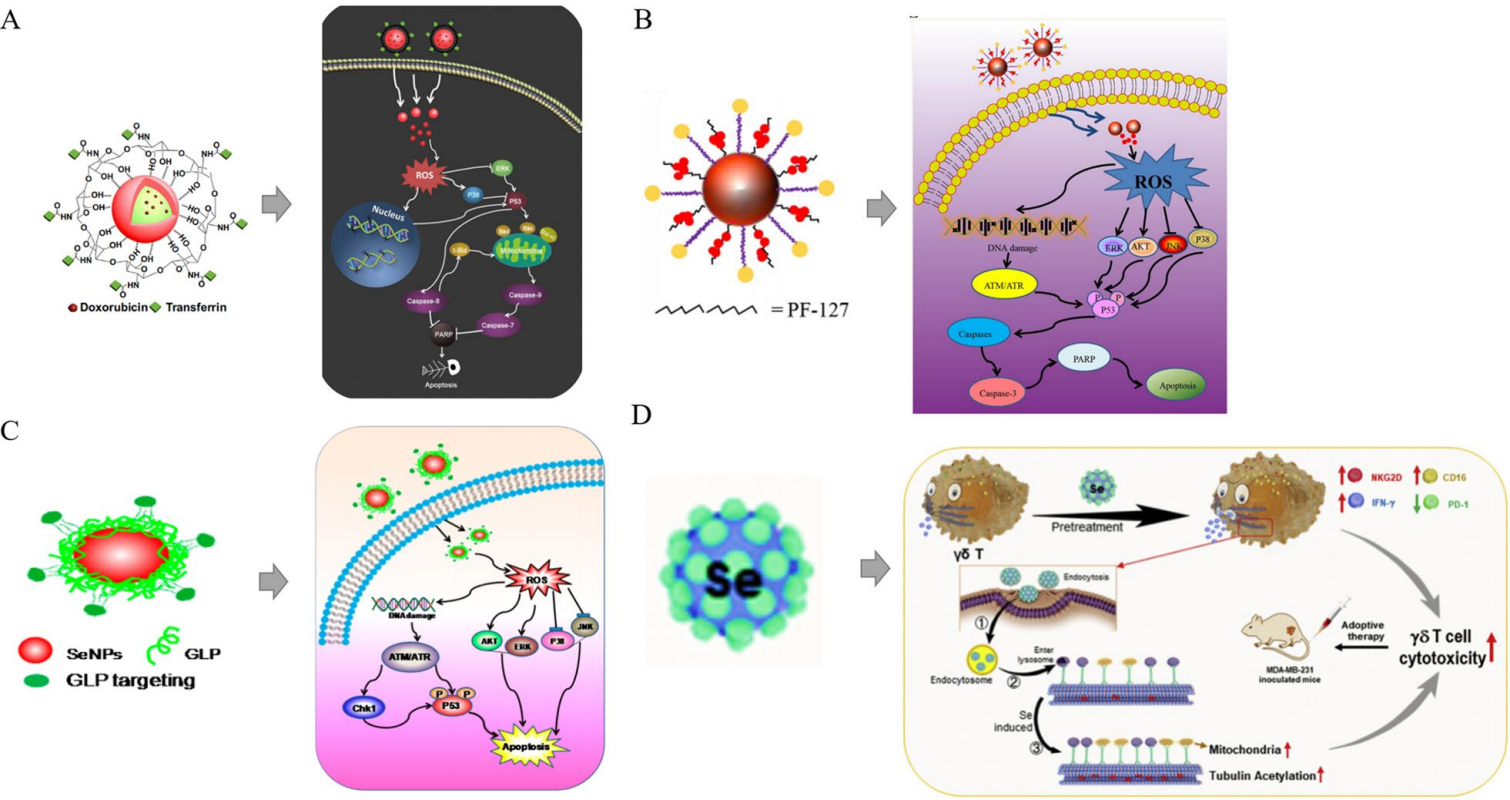
Rational design of different SeNPs for cancer treatments. **A** The internalized Tf-SeNPs trigger the overproduction of intracellular reactive oxygen species (ROS), thereby activating the p53 and mitogen-activated protein kinase (MAPK) pathways and promoting MCF-7 cell apoptosis [47]. Copyright 2013, Elsevier Ltd. **B** Internalized FA-SeNPs trigger ROS overproduction and induce apoptosis of HePG2 cells by activating the p53 and MAPK pathways [48]. Copyright 2015, Elsevier Ltd. **C** After the application of GLP − SeNPs, the p53, MAPK and AKT pathways are activated to promote the apoptosis of U87 cells [49]. Copyright 2014, American Chemical Society. **D** Schematic diagram of SeNP-induced modulation of γδ T cells [50]. Copyright 2019, Elsevier Ltd

In comparison to inorganic and organic compounds (in which inorganic forms are more harmful than organic ones [40–42]), SeNPs have gained considerable attention due to their high bioavailability, strong biological activity, and low toxicity. More suitable items have been created using nanotechnology to guarantee their physiological and therapeutic effects. SeNPs have a wide variety of biological applications, have been developed for dietary supplements as well as therapeutic agents and do not exhibit noticeable side effects in lung cancer. Their impact on decreasing oxidative stress is also well documented [43, 44]. According to the findings of several researchers, SeNPs are beneficial in the chemoprevention of lung cancer [45] as a potential anticancer medication and carrier of anticancer drugs. Tian et al. demonstrated that nanoselenium combined with radiofrequency therapy significantly inhibited lung cancer cell migration and invasion; in addition, the treatment significantly inhibited the expression of the proliferation-related proteins CCND1 and c-Myc and the invasion- and migration-related proteins MMP2 and MMP9, causing lung cancer cells to undergo apoptosis. [46]. Our research group has conducted extensive research on the application of nanoselenium in the treatment of cancer, and the technology for treating lung cancer is relatively mature. SeNPs have gained much attention as potential cancer therapeutic payloads. Transferrin (Tf)-coupled SeNPs were synthesized and used in the present study to enhance cellular uptake and anticancer efficacy as cancer-targeted drugs [47] (Fig. 1A). During the research, it was also found that some SeNPs may lead to drug toxicity and produce adverse side effects for cancer patients. Multidrug resistance is among the biggest challenges in cancer treatment. The uptake of SeNPs was significantly enhanced by folate (FA) surface coupling through nystatin-dependent and clathrin-mediated endocytosis of FA receptors [48] (Fig. 1B). Second, as a surface decoration agent, Gracilaria lemaneiformis polysaccharide (GLP), a polysaccharide of Gracilaria lemaneiformis, stabilizes SeNPs and can be controlled in size*.* GLP − SeNPs showed high selectivity between normal and cancer cells, effectively improving cell uptake and anticancer effects [49] (Fig. 1C). In recent years, immune cell therapy has provided a paradigm for treating malignant tumors. SeNP-pretreated immune cells significantly upregulated the expression of the cytotoxicity-related molecules NKG2D, CD16, IFN-γ and other cells while downregulating the expression of PD-1 in γδ T cells. SeNPs can significantly enhance the antitumor cytotoxicity of immune cells [50] (Fig. 1D). Based on the above studies, the researchers also found that *Polyporus rhinoceros* water-soluble polysaccharide-protein complex (PRW) surface decoration significantly enhanced the uptake of SeNPs by cells through endocytosis. PRW-SeNPs significantly inhibited the growth of A549 cells by inducing apoptosis and G2/M phase arrest. It is possible that PRW interacts specifically with biomolecules and receptors on the cell membrane of cancer cells, thus enhancing the uptake of SeNPs by cells and increasing their cytotoxicity to A549 cells [51]. Additionally, the immunostimulatory action of SeNPs has been verified [52].

The aim of this article was to summarize the recent progress of selenium nanomedicines in the treatment of lung cancer. The introduction will cover several forms of Se, Se compounds, SeNPs, SeNP drugs, and their roles. SeNPs and SeNP-drugs provide a fresh viewpoint on the treatment of lung cancer. In relation to targeted therapies for lung cancer, the article highlights the most significant recent advances in preclinical and clinical research. We also describe current obstacles and offer an overview of possible future perspectives as well as their potential clinical applications in this rapidly developing field.

## Clinical studies of Se in respiratory disease treatment

**Fig. 2:**
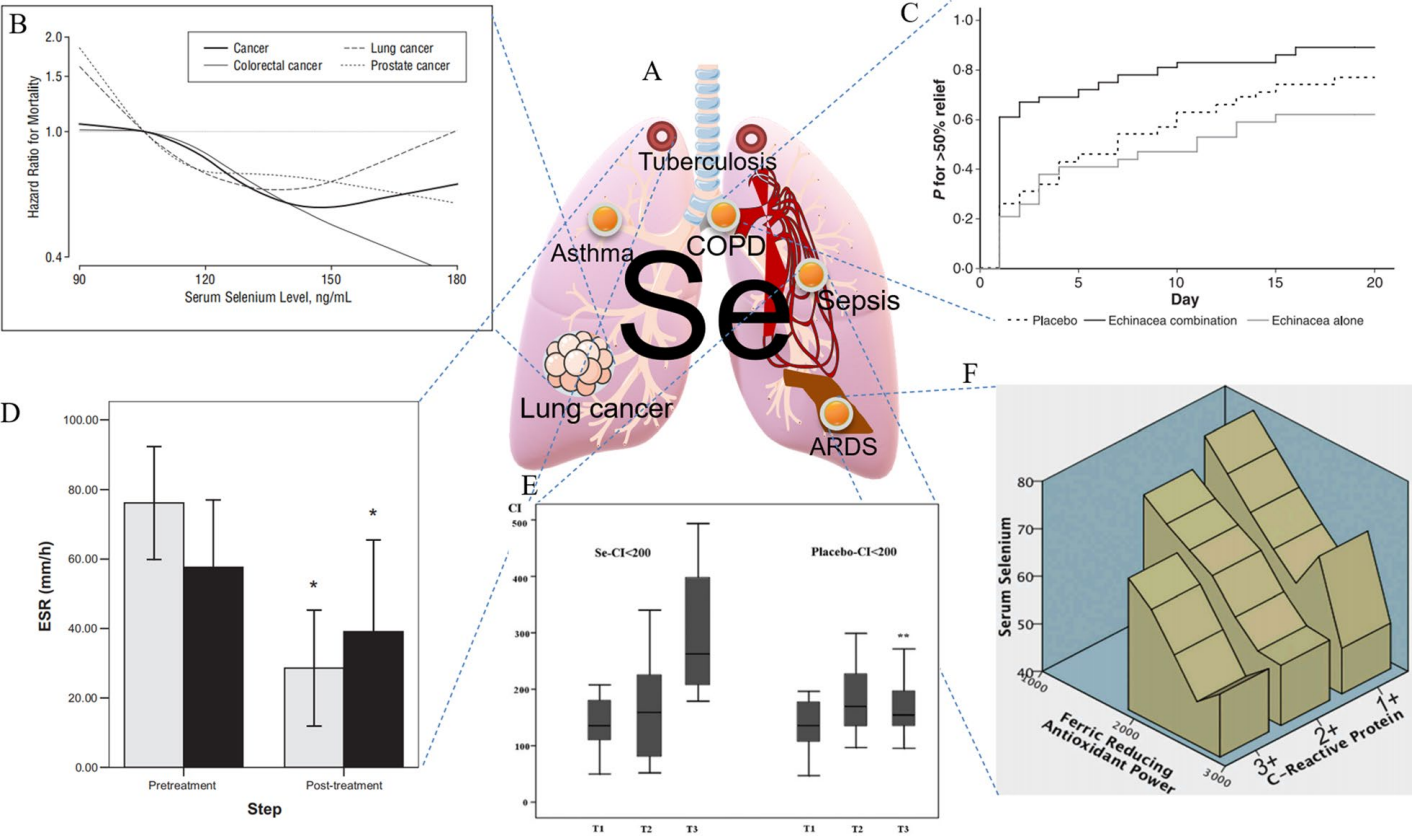
Applications of Se in different respiratory diseases. **A** Application map of Se diseases. **B** Comparison of lung cancer mortality after Se supplementation [66]. Copyright 2008, American Medical Association. **C** Changes in remission rate after Se combined treatment of COPD [56]. Copyright 2010, Blackwell Publishing Ltd. **D** ESR changes in Se after treatment of pulmonary tuberculosis [60]. Copyright 2007, The Authors. **E** Changes in the oxygenation index after Se supplementation [63]. Copyright 2014, The Canadian Society of Clinical Chemists. **F** Three-dimensional description of changes in serum Se, including the effect of bronchoalveolar lavage fluid on the iron-reducing antioxidant capacity (FRAP) of C-reactive protein (CRP) [64]. Copyright 2018, Taylor & Francis

With epidemiological information on lung cancer, researchers have been interested in using Se as a vital and distinctive trace element for treating respiratory disorders (Fig. 2A). Early in 1990, Yu et al. [53] studied the use of Se for preventing lung cancer among Yunnan miners and concluded that Se exhibits an inhibitory effect on lung cancer. In 2002, Reid et al. [54] conducted a follow-up study on Se supplementation and the incidence of lung cancer, showing that Se supplementation could reduce the incidence of lung cancer and was negatively correlated with the incidence of lung cancer (Fig. 2B). Gradually, the application of Se to other respiratory diseases has been reported. A UK study evaluated the effects of Se supplementation on secondary prevention of mild to moderate adult asthma [55]. Isbaniah et al. [56] demonstrated that Se supplementation alleviates exacerbations of chronic obstructive pulmonary disease (COPD) (Fig. 2C). In terms of Se forms, Youssef et al. [57] showed that supplementation with sodium selenite provides a positive effect on the clinical prognosis of low airway respiratory diseases. Further research has shown that Se is mainly used against respiratory disease to promote immune function. Se supplementation enhances immune activity and enhances immune mechanism effects in corticosteroid-dependent asthma [58]. In tuberculosis patients, the immunomodulatory effects of Se were shown to benefit treatments and improve immunity [59], and Se supplementation activated the immune system, improved nutritional deficiency by reducing oxidative stress, and improved the clinical cure rate in patients with tuberculosis [60] (Fig. 2D). In current smokers, Se supplementation reduced the annual decline rate of forced expiratory flow [61]. In another clinical study focused on the relationship between antioxidant nutrients and lung function, Hu et al. [62] found that serum Se exhibited a strong positive correlation with lung function. The same results were found for patients with sepsis. Although research showed that Se supplementation could improve respiratory function, we must also consider that the survival rate of patients was not improved [63] (Fig. 2E). In patients with acute respiratory distress syndrome (ARDS), Mahmoodpoor et al. also found that Se supplementation could moderately regulate the inflammatory response and improve respiratory function [64] (Fig. 2F). In addition, if Se is used improperly, many side effects can occur. For example, Karp et al. [65] have shown that long-term, large amounts of Se supplementation can lead to adverse effects, such as dyspnea, weakness, nail changes, and dry skin.

Although the Se use of is increasing, its application and the clinical studies of Se are not comprehensive, and the forms, methods and means of Se use have not been summarized. Many studies have shown that we lack effective low-drug Se. Recently, it has been encouraging to see that many Se-based drugs, especially SeNP-based drugs, have been developed for respiratory diseases, with a focus on lung cancer.

## Se forms and their use in lung cancer treatment

**Fig. 3:**
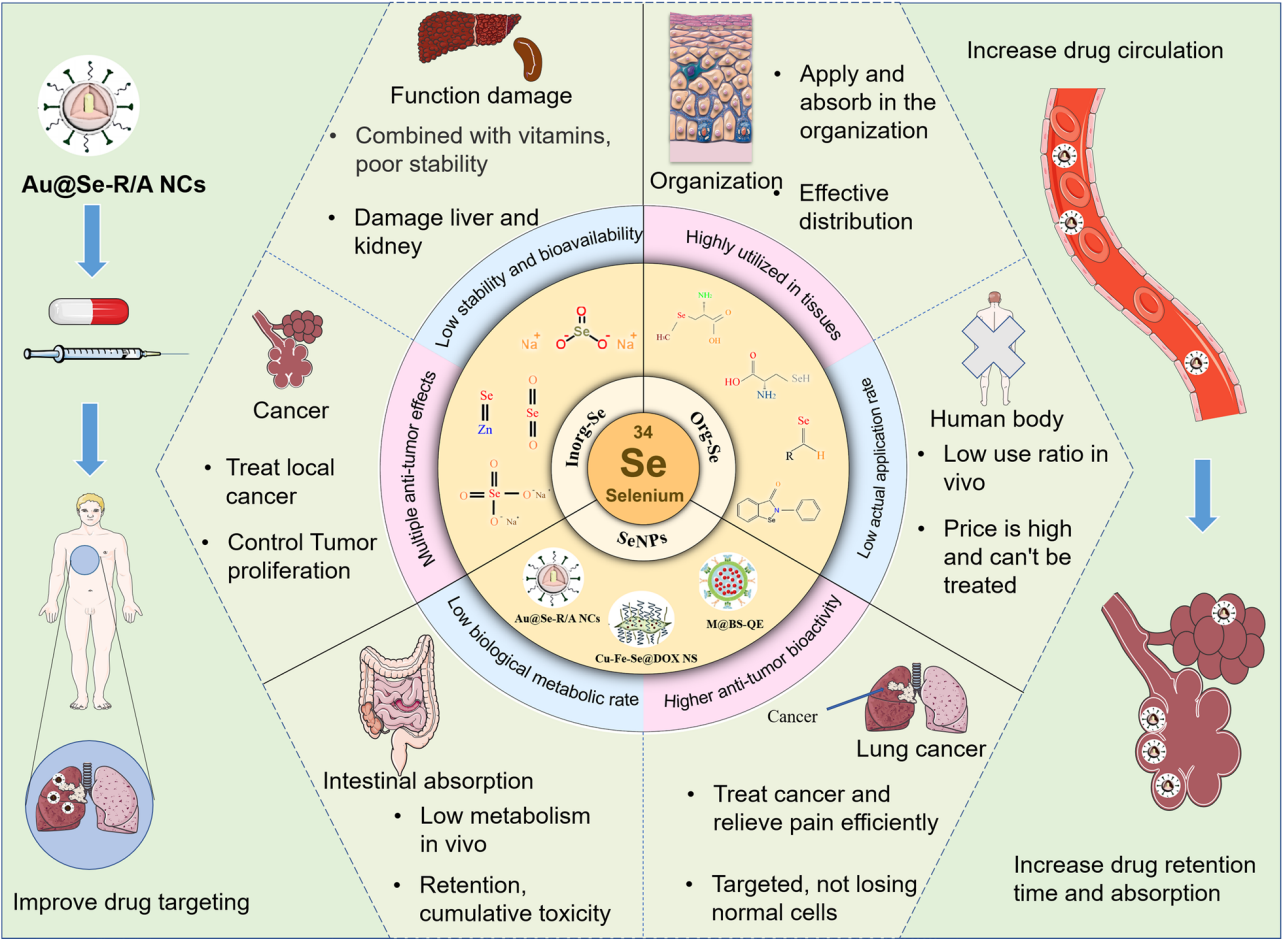
Different Se forms used in lung cancer treatments. The distinguishing degree and characteristics of the three forms of Se are shown [46, 71–77]. Copyright 2020, Tian, Wei, Zhang and Xu. Copyright 2020, Alkie et al. Copyright 2008, Elsevier B.V. Copyright 2001, Oxford University Press. Copyright 2021 by the authors. Copyright 2020, Chen, Li, Cong, Yu, Zhu, Barba, Marszalek, Puchalski and Cheng. Copyright 2000, Springer-Verlag New York Inc. Copyright 2019, Elsevier B.V

In our daily lives, different Se compounds, mostly Inorg-Se and Org-Se, can be obtained from food. Se is mostly found in organic compounds, such as selenomethionine, as well as in inorganic compounds, such as selenite and selenate. Typically, they are transformed into the metabolite hydrogen selenide (H_2_Se), which is the building block for selenophosphate. Glutamylmethylselenocysteine can also produce methylselenol (CH_3_SeH). It is possible to change H_2_Se and CH_3_SeH into one another. In contrast to natural forms of Se, compounds, such as methylseleninic acid (MSA) [67], can be synthesized in the laboratory. Different Se molecules enter the metabolic pathway at various times through the actions of various enzymes. Selenoproteins always have a biological function in cells synthesized from selenophosphate, but some effects and/or mechanisms of Se are specific to certain forms of Se [68–70]. In addition, with the development of technology, more stable and nontoxic SeNPs have been synthesized by nanotechnology (Fig. 3).

### Inorg-Se in tumor treatment

Most of the early studies concentrated on Inorg-Se and have provided considerable evidence demonstrating the in vitro antitumor effects of Se. Inorg-Se agents exhibit a cytotoxic effect, which can directly kill cancer cells and inhibit their aberrant proliferation [78]. Inorg-Se also show a differentiation-promoting effect on cancer cells, which can result in reducing the invasiveness of tumors and ameliorating the prognosis of lung cancer patients [79]. In addition, Inorg-Se was shown to improve the response to chemotherapy [80] and has the ability to reduce the systemic toxicity of cancer chemotherapeutic drugs [81–83]. Other studies have shown that Se not only combats renal and cardiac toxicity by increasing intracellular superoxide dismutase (SOD) and glutathione peroxidase (GPX) levels and activities to inhibit peroxide-induced nuclear factor kappa beta (NF-κB) activation but also stimulates the production of immunoglobins and antibodies, which could improve the systemic immunity of patients [73, 84].

Overall, Inorg-Se is a promising and inexpensive antitumor agent with multiple antitumor effects, as confirmed by laboratory studies. However, Inorg-Se must bind to organic ligands in the gut before it can be absorbed by the human body, but it can also easily bind to vitamins in the body [85]. As many factors compete with Se for organic ligands in the intestinal tract, the absorption rate, stability and bioavailability of Se are low [74]. In addition, Inorg-Se is relatively toxic; if the human body overdoses locally, it can cause irreversible damage [72].

Thus, considering the good antitumor efficacy of Se but limited employment of Inorg-Se, researchers have worked to overcome the shortcomings of Inorg-Se to develop Org-Se and SeNPs. For example, Inorg-Se can be converted to Org-Se by natural transformation (through biochemical mechanisms in the body through plants, animals and microorganisms) and artificial synthesis (using chemical methods) [86, 87], SeNPs are prepared by nanotechnology [88]. Consequently, Se products featuring low toxicity and good stability have been synthesized.

### Org-Se in tumor treatment

Org-Se can be stored easily in tissues, absorbed, and rapidly utilized by the human body after absorption*.* Org-Se has been shown to be associated with four types of cell death pathways, including cell cycle arrest, autophagy, apoptosis and necrocytosis [89], indicating its potential anticancer application. One of the best-known Org-Se compounds, ebselen—an Org-Se compound with antioxidant and anti-inflammatory properties—is a GPX mimetic and excellent peroxynitrite scavenger. Ebselen has a Se-N bond as a stimulated GPX active site, as well as a protecting Se-C bond structure to prevent Se atom release and maintain relatively low systemic toxicity compared to that of Inorg-Se. With regard to its antitumor effect, ebselen mainly acts by inhibiting thioredoxin activity in tumor cells, regulating downstream pathways and inducing tumor cell apoptosis [90]. In addition, similar to Inorg-Se, Org-Se has the ability to reduce the systemic toxicity of cancer chemotherapeutic drugs. Hu et al. observed reduced nephrotoxicity and leucopenia in solid tumor patients receiving cisplatin accompanied by seleno-kappacarrageenan, an Org-Se agent used clinically [91].

Based on previous studies, current Org-Se research in our group focuses mainly on antitumor targets and mechanisms. On the one hand, a new target has been found by using chemical biology and other techniques, which confirmed the mechanism of interaction among p53, Org-Se, and TrxR targets [92]. On the other hand, the sensitizing effect of Org-Se on RT and chemotherapy has been evaluated extensively [93]. These conclusions may enrich our knowledge of the antitumor effects of Org-Se.

Org-Se generally exists in the form of selenomethionine, which is involved in protein synthesis and is easily stored, absorbed and highly utilized in tissues. At the same time, Org-Se exhibits reduced toxicity and better biocompatibility than that of Inorg-Se, although unavoidable problems, such as potential systemic toxicity, remain [75]. In terms of safety, Org-Se does not have a very strong advantage over Inorg-Se, and research shows that both forms are subchronic toxic agents; moreover, the Org-Se production process is complex and costly, and the practical applications are fewer [76]. To solve this problem, nanosized Org-Se can be prepared by using nanotechnology to reduce toxicity and improve the safety of Org-Se [94, 95]. Therefore, virtually nontoxic and more potent SeNPs have emerged into the medical field and have become a hot topic in the cancer treatment field.

### SeNPs in tumor treatment

As noted already, nanotechnology has greatly suppressed the potential toxicity associated with Inorg-Se and Org-Se while improving the targeting of drugs and realizing the development of personalized therapy. In nanomedicine, nanoparticles (NPs) have emerged as attractive carriers for intracellular delivery of drugs [96]. Due to their accumulation in tumor tissue, nanoparticles have the potential to kill tumor cells locally at high concentrations, increase the curative effect, and reduce the side effects and toxic effects of drugs. Therefore, NPs can be used to create special drug carriers. An analysis of patents and literature showed that the materials used as drug carriers mainly included metal NPs [97], inorganic nonmetallic NPs [98], biodegradable polymer NPs [99], and bioactive NPs [100]. However, the cytotoxic effects of a large number of inorganic NPs have been evaluated in cancer cells, and SeNPs were found to show the greatest potential as new antitumor drug candidates [46].

Compared with Inorg-Se and Org-Se, SeNPs were shown to have lower cytotoxicity and higher antitumor bioactivity [77]. The antitumor mechanism of NPs, particularly SeNPs, has been widely proposed, including inhibiting cell proliferation, inactivating carcinogens, stimulating the immune system and promoting cell apoptosis. The latter is the major mechanism in their antitumor effect at the molecular level [48, 77, 101, 102]. It has been well documented that oxidative stress and the formation of ROS are the major signaling pathways mediating the cytotoxicity induced by SeNPs, in which ROS can be modulated to induce intrinsic apoptosis by modifying the activity of enzymes involved in cell death pathways [48, 101, 102]. SeNPs appear to be more effective at scavenging free radicals than other forms of Se [103]. SeNPs have also been found to exhibit extraordinary effects in areas such as combined chemotherapy, radiosensitization, resistance to chemotoxicity, immunotherapy, and photothermal therapy (PTT) of cancers [104–107]. For example, Ru-MUA@Se [108], CFS@DOX NSs [109], UCNFs-Bi_2_Se_3_ [110], etc., can be applied to cancer diagnostic imaging; PEG-SeNPs [111], PHD_2_ [112], RBCs@Se/Av[113], FA@SeNPs [114], Se@MUN [115], TeSe [116], SeNPs@LNT [18], etc., can be applied to cancer treatment. They may act either directly or through radiochemotherapy sensitization and immunotherapy to play an anticancer role.

Overall, this review elaborates on the cutting edge of SeNPs in the field of lung cancer therapy.

## Functional design and synthesis of Se-based nanomedicine for lung cancer theranostic applications

### Synthesis and application of SeNPs in the treatment of lung cancer

**Fig. 4:**
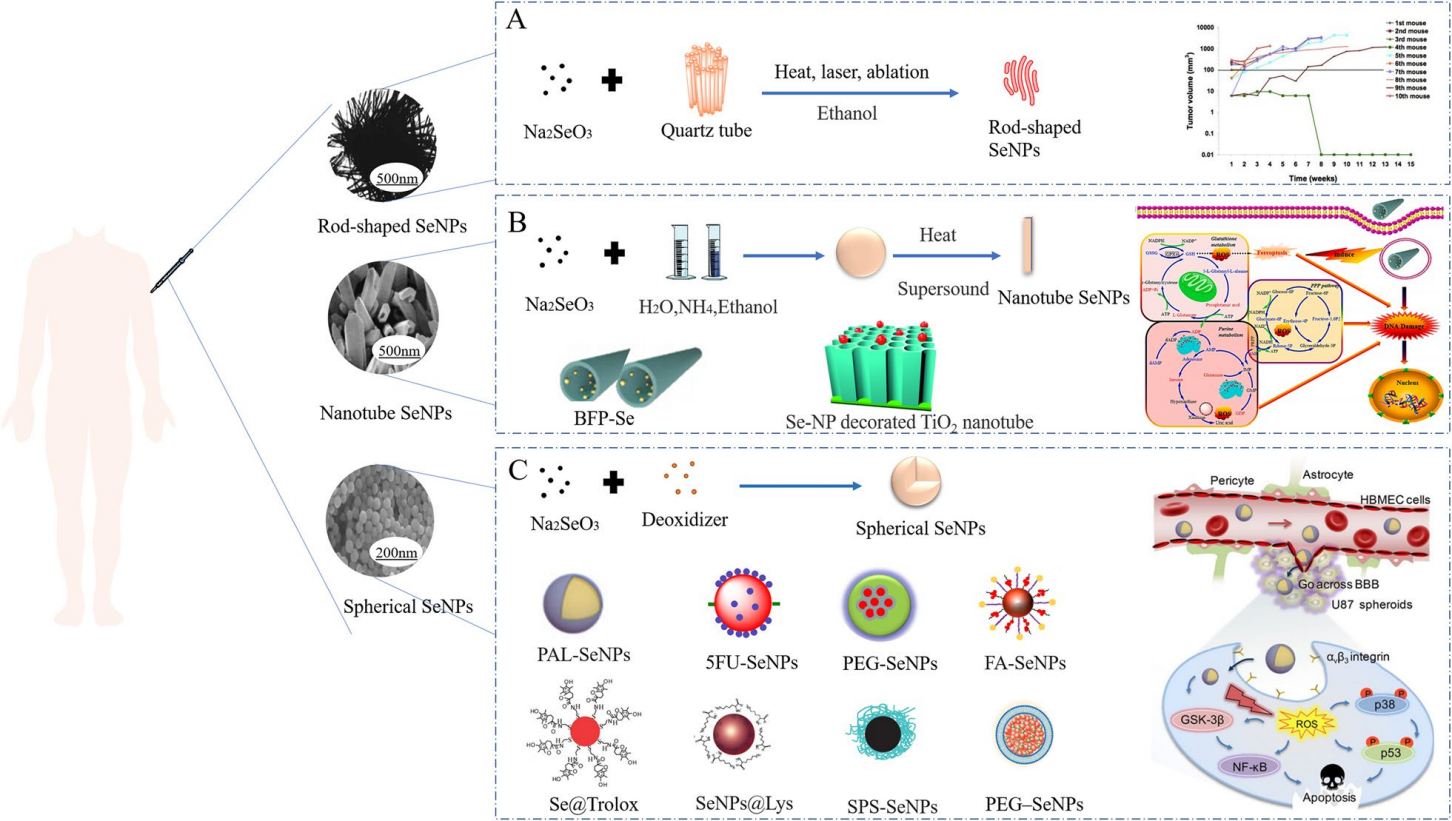
Morphology of different SeNPs used in cancer treatment and their biological application. **A** Sodium selenite and quartz tubes were treated with alcohol by heat, laser, and ablation to synthesize rod-shaped SeNPs [117, 118]. Copyright 2002, Elsevier Science B.V. Copyright 2018, IMSS. **B** Nanotube SeNPs were synthesized with sodium selenite, water, ammonia and ethanol by heating and sonication [119–121]. Copyright 2004, American Chemical Society. Copyright 2022, Elsevier B.V. Copyright 2019, Bilek et al. **C** Synthesis of spherical SeNPs from sodium selenite and potato extract [48, 104, 107, 113, 122–126]. Copyright 2015, Elsevier Inc. Copyright 2012, American Chemical Society. Copyright 2015, Elsevier B.V. Copyright 2006, The Royal Society of Chemistry. Copyright 2003, Regional SOCIETY OF CHEMISTRY. Copyright 2013, Royal Society of Chemistry. Copyright 2018, WILEY‐VCH. Copyright 2018, Royal Society of Chemistry

The size and morphology of SeNPs can affect their biological activity and uptake capacity of cells [127]. Therefore, it is very important to select the appropriate method for preparing the desired nano size and morphology of SeNPs (Fig. 4). In view of this, SeNPs can be synthesized with the following methods: physical synthesis, biological synthesis, and chemical synthesis (Table 1).

**Table 1:** Characteristics of the three methods of SeNP synthesis for lung cancer

Production method	SeNPs characteristics	Advantages	Disadvantages	References
Physical synthesis	• γ-radiation • Microwave radiation • Pulsed laser ablation in liquids (PLAL)	• Rapid and uniform production • Environmentally friendly high physical properties • Controlled and not easily contaminated by chemical reagents	• Low efficiency • Low yield • High energy consumption • Easy sample contamination • Uneven particle size	• [128–130]
Biological synthesis	• Biological synthesis uses plant extracts, fungi and bacteria to synthesize biogenic SeNPs • Microbial synthesis refers to the reduction of other forms of Se to SeNPs through microbial metabolism products	• Safe, hypotonicity • Recyclable • Environmentally friendly • High stability	• Conditions are very strict, not very simple • Poor quality control • SeNPs surface composition unknown	• [131–136]
Chemical synthesis	• Through REDOX reaction	• Simple process • Controllability • Stable chemical structure • Diameter of uniform	• High cost of production • Presence of toxic byproducts	• [50, 137–140]

#### Physical synthesis and application of SeNPs

SeNPs have been prepared by physical synthesis using γ-radiation and microwave radiation [128], and pulsed laser ablation in liquids (PLAL) is a novel preparation technique for preparing pure naked SeNPs [129]. For example, Guisbiers et al. obtained SeNPs with a particle size of 115 ± 38 nm by pulsed laser ablation of pure Se pellets immersed in deionized water [130]. Physical synthesis can be used to prepare SeNPs with environmental benignity and high physical properties. However, there are some problems, such as high energy consumption, easy sample contamination, and uneven particle size.

#### Biological synthesis and application of SeNPs

In biological synthesis, plant extracts, fungi and bacteria are used to synthesize biogenic SeNPs. Microbial synthesis refers to the reduction of other forms of Se to SeNPs through microbial metabolism products [141, 142]. As a secondary metabolite and function of the plant extract, phenols, flavonoids, amines, alcohols, proteins and aldehydes are involved in reducing Se to SeNPs [143]. For instance, Fesharaki et al. cultured *Klebsiella pneumoniae*-containing SeNPs and released SeNPs with a particle size of 245 nm [142]. Biological synthesis is safe, environmentally friendly and recyclable. However, the production method and conditions are very strict and not very simple. Several parameters and steps remain to be optimized in the biosynthesis of SeNPs.

#### Chemical synthesis and application of SeNPs

The chemical synthesis of SeNPs is prepared through the REDOX reaction, in which selenate or Se-dioxide is often used as a source of Se. Vitamin C, sodium sulfite (Na_2_SO_3_), and sodium thiosulfate are used as reducing agents, stabilizers can be added appropriately during the process, and SeNPs can be successfully prepared. In 2010, Langi et al. reported the first chemical synthesis of SeNPs using sodium selenosulfate and 3-methylimidazolium sulfate in an ionic liquid-assisted manner [137]. Under the action of a polyvinyl alcohol stabilizer, sodium selenosulfate was used as the precursor of Se to synthesize 76–150 nm SeNPs. In addition, the molecular structure prevents SeNPs aggregation and improves its stability. Zhang et al. prepared spherical SeNPs with a particle size of 36.8 ± 4.1 nm using selenite as the Se source, ascorbic acid as a reducing agent, and β-lactoglobulin (Blg) as a stabilizer [144]. This also indicates that the reaction in ionic solution needs the addition of a certain amount of stabilizer, usually a polysaccharide such as chitosan (CS) [139], spiral algae polysaccharide (SPS) [126], polysaccharide-protein complex (PSP) [145], or sodium formaldehyde sulfonate (SFS) [146] stabilizer solution. Polysaccharides extracted from seaweed can improve the stability of SeNPs [147]. Cordyceps exopolysaccharides (EPS) [148], acacia gum, or carboxymethyl cellulose are also used as polysaccharides. Lentinan, extracted from the medicinal basidiomycetes *Lentinula edodes* [149], can not only be used as a stabilizer but also reduces Inorg-Se and Org-Se compounds to SeNPs. Chen et al. found that polysaccharides extracted from seaweed wakame can enhance the stability of SeNPs and form monodisperse spherical SeNPs for treating A375 human melanoma cells [147].

In view of the dual effects of lentinan, we synthesized SeNPs named SeNPs@LNT that can transform cold malignant pleural effusion (MPE) into hot MPE using LNT as the polysaccharide. Various biophysical methods, including electron microscopy, were used to characterize SeNPs after they were synthesized. As a food additive, monodisperse spherical SeNPs are very stable in solution. Chemosynthetic materials are readily available and easy to use and can be synthesized at the atomic or molecular level in sizes, shapes, and crystal types that are easily controllable. However, the high cost of production and the presence of toxic byproducts have limited the development of new methods for the synthesis of nanoparticles.

### Synthesis and application of SeNP composites in the treatment of lung cancer

Nanocomposite materials are composed of nanosized inorganic particles, metals, semiconductors, rigid particles, etc., which are prepared by appropriate preparation methods. Recent research has shown that nanocomposites, including Se nanocomposites, can have medical applications, such as in cancer [150]. SeNPs exhibit unique properties, and as a drug, they possess strong penetration properties and cause little damage to the body. The Se nanocomposites currently studied include porous Se@SiO_2_ nanocomposites, Cu_2_-XSe nanocrystals coated with silica oxidized to Se quantum dots and PVP etched to form porous structures [151], which effectively inhibit the proliferation of cancer cells through the ROS-mediated mechanism. Au@Se core–shell nanostructure: a seed-mediated method was used to synthesize Au NRs after the formation and conjugation of Se shells [152]. Combined with X-ray therapy, Au NRs can induce cell apoptosis by altering the expression of p53 and dna damage genes, triggering the excessive production of intracellular ROS and greatly improving the anticancer effect. Se dioxide (SeO_2_) NPs and Se dioxide/titanium dioxide nanocomposites (Se/Ti (I), (II) and (III)) can also treat cancer [153]. Nanocomposites exhibit good biosafety due to the controlled release of Se, which not only ensures a beneficial effect but also reduces toxicity [151], and the prospects for their successful application are very promising.

### Synthesis and application of Se-based two-dimensional nanomaterials for the treatment of lung cancer

**Fig. 5:**
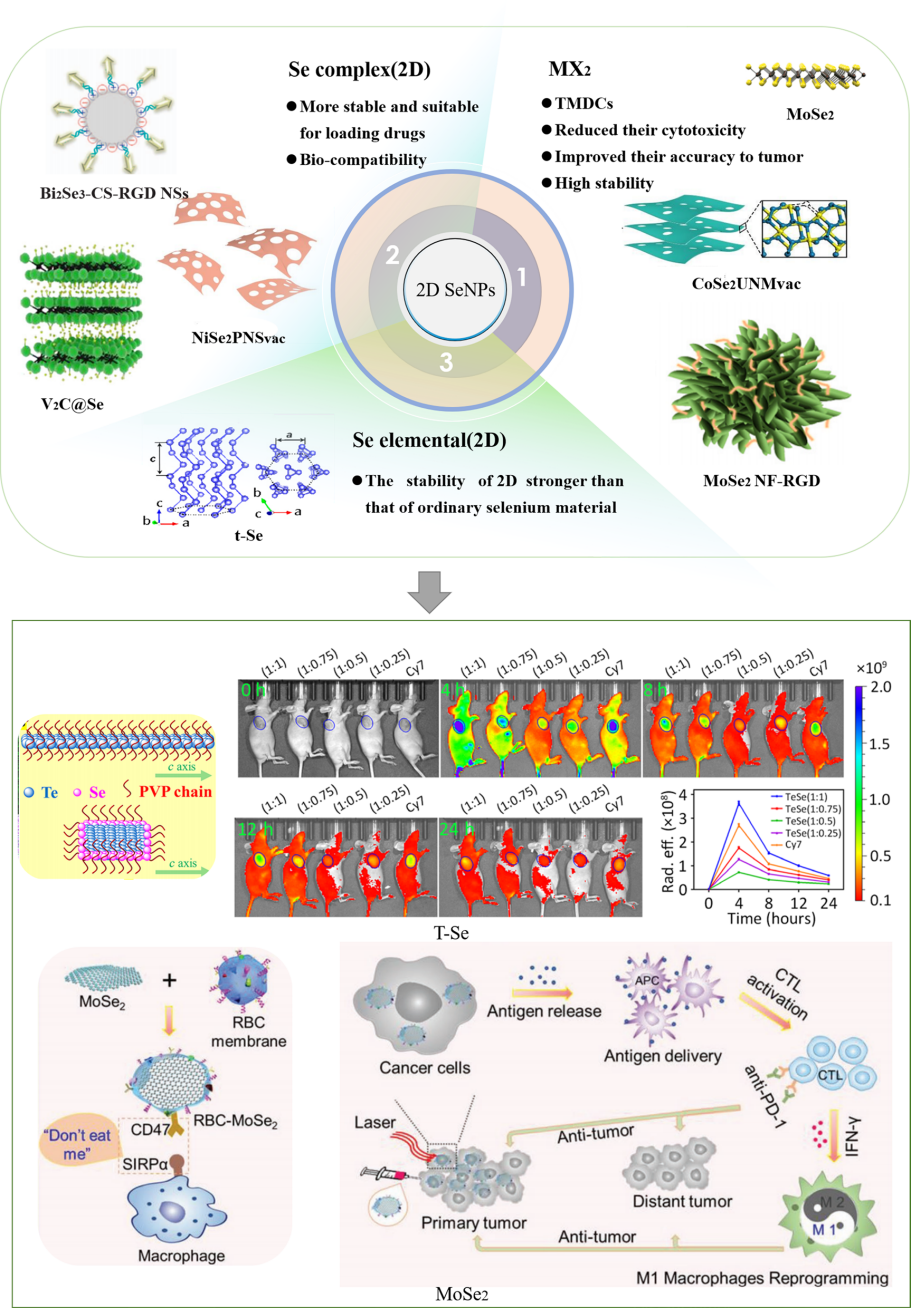
Structure of 2D SeNPs for cancer. 2D SeNPs are divided into three categories (MX_2_, Se elemental, and Se complex) because their structures and characteristics are different [116, 161–169]. Copyright 2021, American Chemical Society. Copyright 2016, WILEY‐VCH. Copyright 2019, Royal Society of Chemistry. Copyright 2018, American Chemical Society. Copyright 2022, Elsevier B.V. Copyright 2020, The Author(s). Copyright 2017, The Author(s). Copyright 2019, WILEY‐VC. Copyright 2022, Elsevier Ltd. Copyright 2020, The Authors

In recent years, with the progress of science and technology, two-dimensional (2D) nanomaterials have emerged, expanding from the physical field to the biological field [154, 155]. A 2D nanomaterial is a type of nanomaterial that is freestanding and sheet-like in shape, with a high ratio of lateral size to thickness [156]. A tremendous amount of interest has been generated by the unique nanosheet structures, large surface areas, and extraordinary physiochemical properties [157]. The large surface areas of 2D nanomaterials make them highly effective nanoplatforms for drug delivery. Phototherapy and RT of cancer can be enhanced by utilizing the unique optical and/or X-ray attenuation properties. 2D nanomaterials can also be engineered to serve as multimodal tumor imaging probes by integrating them with other functional nanomaterials or utilizing their inherent physical properties [158]. Jiet et al. prepared high-quality ultrathin boron nanosheets, which have great prospects in cancer diagnostic imaging and image-guided drug delivery [159]. Xie et al. researched a 2D SnS-based dual-function nano-PTT platform that proved to be effective against several cancer cell lines and xenograft tumors [160]. At present, 2D SeNPs are also the subject of intense research efforts. In the following section, we introduce 2D SeNP materials, which can be divided into the following categories: MX_2_, Se elemental (2D), and Se complex (2D) (Fig. 5).

#### Synthesis and application of MX_2_ for the treatment of lung cancer

Monolayer transition metal dichalcogenides (TMDCs) have layered structures similar to graphite and have attracted extensive attention because they are naturally abundant, and some TMDCs are semiconductors with considerable band gaps [170]. Due to the diversity of chemical composition and structural phases, TMDCs exhibit abundant electrical properties both in terms of band structure characteristics (metallic properties and insulation) and the appearance of related and topological phases. A single layer of 2D TMDCs (the generalized formula is MX_2_, in which M is a transition metal of the 4–10 group and X is a type of copper) exhibits a variety of chemical properties [171]. Wang et al. developed a novel photothermal nanocarrier, polydopamine-coated molybdenum selenide, which can load the anticancer drug DOX [172]. This not only enhanced the photothermal effect of molybdenum selenide (MoSe_2_) nanosheets but also reduced their cytotoxicity and improved their accuracy in the tumor. Pan et al. prepared Gd^3+^-doped MoSe_2_ (MoSe_2_(Gd^3+^)-polyethylene glycol (PEG) nanosheets [173]. The MoSe_2_(Gd^3+^)-PEG nanosheets, which exhibit high stability in physiological solution and show no obvious toxicity in vivo, can be used as a contrast agent for photoacoustic imaging (PAI). MoSe_2_(Gd^3+^)-PEG combines therapeutic and imaging capabilities to achieve cancer therapy. Dong et al. prepared Rh_3_Se_8_ NPs that exhibit many characteristics, such as high photostability, negligible adverse inflammatory effects, and low long-term toxicity; these NPs show great potential for bioimage-guided efficient photonic cancer thermotherapy for nanosystems [172]. 2D SeNPs, especially MX_2_, are more efficient in the space structure, effectively carrying and releasing drugs while reducing toxicity to a lower level, and future studies may find that these NPs have more advantages and wider applications.

#### Synthesis and application of Se (2D) for the treatment of lung cancer

The stability of 2D nanomaterials is similar to that of carbon nanotube-based nanomaterial systems and stronger than that of ordinary Se materials[173]. Qin et al. studied the anisotropic mechanical properties of individual 2D trigonal Se (t-Se) nanosheets [161]. Studies of the structure and properties of 2D t-Se have laid a good foundation for its application in biology. With the development of science and technology, 2D t-Se has become a new member of the 2D semiconducting nanomaterial family.

#### Synthesis and application of the Se complex (2D) for the treatment of lung cancer

In addition to the above two categories, 2D SeNPs also include the Se complex, which is more stable and suitable for loading drugs. Multifunctional 2D Bi_2_Se_3_ nanosheets can be used for antibacterial and anti-inflammatory treatment of bacterial infections [174], although the antitumor effects remain to be developed. Nevertheless, Chen et al. studied the stability and biocompatibility of the newly synthesized Se tellurium [116], which exhibits obvious tumor-targeting and antitumor effects.

More importantly, because Se can affect the TIME, 2D SeNPs possess characteristics that other 2D nanomaterials do not have, which can enhance immune cells and affect the TIME. In the near future, it is anticipated that such research may be greatly expanded, and there are high expectations for the treatment of cancer, especially lung cancer.

## SeNPs for lung cancer diagnosis and imaging

SeNPs can not only be used as carriers to transport drugs to the tumor site but also as probes and contrast agents for the diagnostic imaging of lung cancer to improve the level of modern medical diagnosis. A variety of compounds have been approved for clinical use and imaging purposes [175]. Current imaging techniques used include fluorescence imaging (FRI), MR imaging (MRI), CT and PAI.

### Application of SeNPs in FRI

**Fig. 6:**
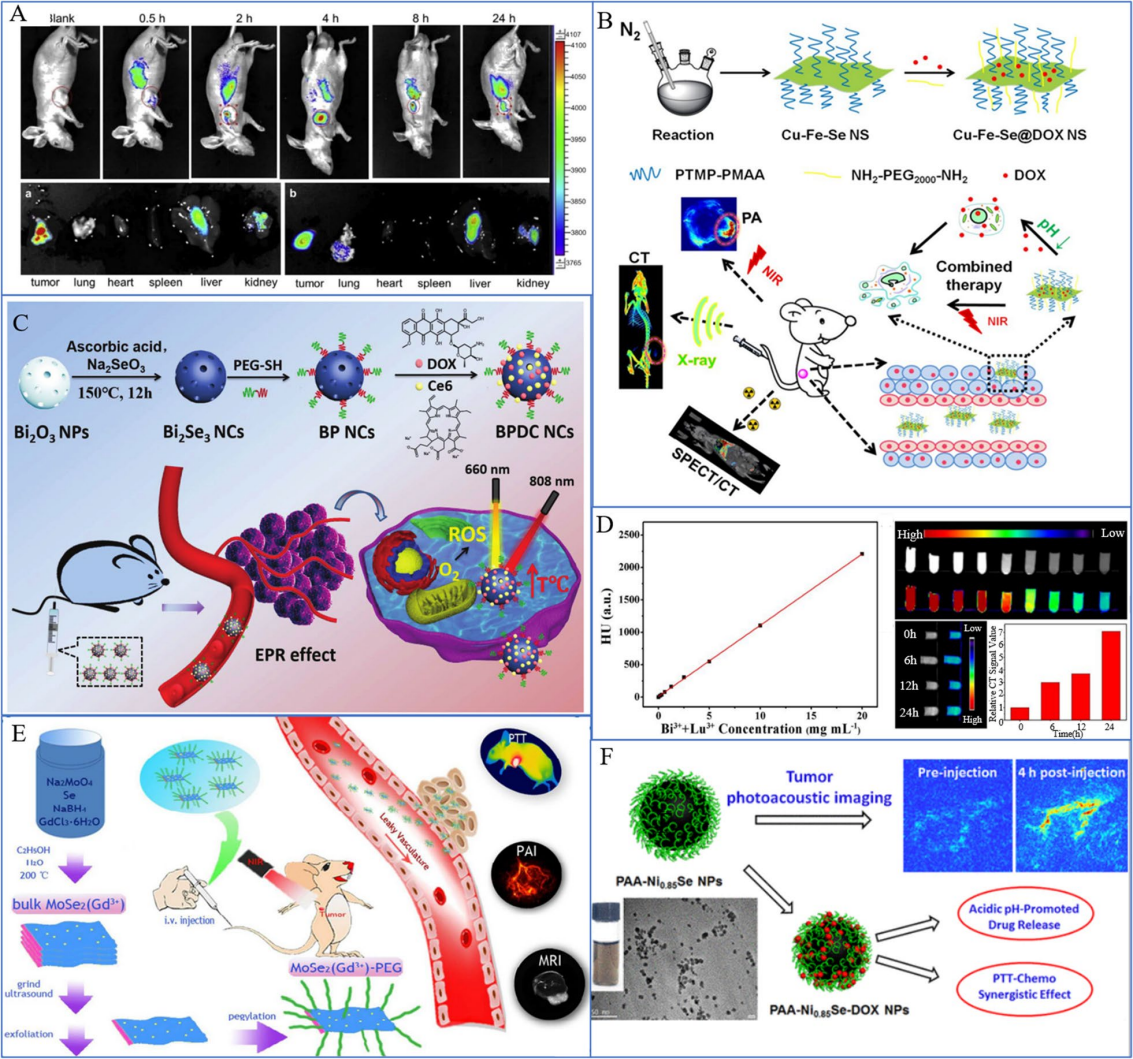
SeNPs for cancer diagnosis and imaging. **A** In vivo imaging of tumor-bearing mice and in vitro FRI of each tissue at different times after Ru-MUA@Se injection [108]. Copyright 2013, Elsevier Ltd. **B** (CFS@DOX NSs as a diagnostic probe for CT imaging and combined chemotherapy/light therapy [109]. Copyright 2018, American Chemical Society. **C** BPDC NCs represent a multifunctional and versatile biomedical platform for tumor diagnosis by using FRI [176]. Copyright 2019, Royal Society of Chemistry. **D** CT image of A549 cells incubated with UCNPS-Bi_2_Se_3_ nanoheterozygotes [110]. Copyright 2019, Wiley‐VCH. **E** MR-pegylated MoSe2(Gd^3+^) injected into mice can diagnose cancer by MR [171]. Copyright 2018, Royal Society of Chemistry. **F** PAA-Ni0.85Se-DOX NPs achieve photothermal-chemical cancer therapy through PAI [177]. Copyright 2017, American Chemical Society

FRI refers to the visualization of colorless and transparent cells, organs, or specific proteins that cannot be observed directly by fluorescent labeling reagents or fluorescent antibodies to observe and analyze the morphology or structure and life activities of cells and aid in the differential diagnosis of cancer [178, 179]. In preclinical research, FRI has become among the most commonly used imaging tools [180]; it is mainly used to diagnose tumors (including lung cancer) by fluorescence probes [181]. In addition, FRI can be used in the clinical diagnosis of disease by labeling tumor cells [180]. In recent years, the application of SeNPs has gradually developed. Since the NPs lack fluorescence characteristics but have an unloading effect, they can be modified with other substances with photosensitizer properties or those with high absorption of light properties to form complexes and play a photosensitive FRI role. Some examples include Ru-MUA@Se and Bi_2_Se_3_@PEG/DOX/Ce_6_. Sun et al. prepared bright and photostable thiol-modified SeNPs with the attached photosensitizer Ru(II)-polypyridine complex and conducted experiments at various tumor sites through FRI technology to identify different types of tumors [108] (Fig. 6A). Sun et al. concluded that Bi_2_Se_3_@PEG/DOX/Ce_6_ revealed the in vivo biological distribution through externally stimulated FRI, providing accurate diagnostic information for tumor treatment [176] (Fig. 6C). These results indicate that SeNPs play an important role in the fluorescence diagnosis of various cancers, including lung cancer.

### Application of SeNPs in MRI

MR is a biological magnetic spin imaging technology that, in computer technology, uses the nuclear magnetic resonance principle through detecting electromagnetic wave imaging [182, 183]. Since 1996, pulmonary functional MR techniques have been applied continuously in the clinic [184, 185]. Currently, researchers are combining nanotechnology with MR imaging applications to improve diagnostic tumor technology. The reason why NPs can be used as contrast agents for MR imaging is that the required NPs are limited—magnetic NPs, such as SeNPs with a TMDC structure and iron diselenide (FeSe_2_) NPs. Pan et al. reported that MoSe_2_(Gd^3+^)-PEG with a TMDC structure can be used as a T1-weighted MRI contrast agent to diagnose tumors in vivo [171] (Fig. 6E). Fu et al. developed magnetic FeSe_2_ NPs and modified them with PEG to form pegylated FeSe_2_ NPs with a high R2 relaxation rate and strong NIR absorption rate, which can be used for MRI [186]. Magnetic nanomaterials are being further developed and will soon have many applications in medical diagnostics.

### Application of SeNPs in CT imaging

CT is a relatively new clinical diagnostic method that can distinguish the difference in X-ray absorption capacity and transmittance between different tissues, input the measurement data into the computer, and reconstruct the fault plane image after the computer processes the data [187, 188]. When mixed with other materials, the negatively charged surface and multifunctional groups of composite nanomaterials can play a defining role in CT imaging, even if SeNPs alone do not have an isotope labeling function. Cu-Fe-Se ternary nanosheets have been examined by Jiang et al., and their surface and multifunctional groups allow for CT imaging diagnosis [109] (Fig. 6B). The UCNP-Bi_2_Se_3_ nanocomplex synthesized by Zhao et al. showed efficient upconversion luminescence (UCL) and reasonable CT imaging ability, highlighting the efficiency of this approach in UCL imaging and PTT [110] (Fig. 6D). Of course, whether the SeNPs can be used remains unclear.

### Application of SeNPs in PAI

PAI uses substances with optical absorption to target and accumulate at the lesion site, converting the energy of the pulsed laser into heat, thus causing thermal expansion to generate ultrasonic signals, and constructs images by detecting such signals [189, 190]. Due to nickel selenide's distinctive electron configuration and relatively high catalytic activity, Wang et al. reported a multifunctional theranostic agent made of ultrasmall poly(acrylic acid)-functionalized Ni_0.85_Se NPs (PAA-Ni_0.85_Se NPs) [177]. This agent was successfully used in PAI (Fig. 6F). Although less well researched, other combinations are also being investigated in this area.

## Application of SeNPs in traditional treatment of lung cancer

### Application of SeNPs to directly kill tumor cells

The biological action of Se has been researched thoroughly since the late 1980s, which has led to the rapid advancement of Se-based agents. Inorg-Se agents, such as selenic acid, Se oxide, and selenite sodium, were the focus of the majority of early studies. These studies demonstrated the strong antitumor activity of Se, and in September 2003, the US Food and Drug Administration (FDA) officially recognized Se as an antitumor agent, reiterating the agent's efficacy. The primary area of our study is Se nanomedicine. Se not only exhibits fewer side effects and is biocompatible but can also target tumor cells directly. By preventing the creation of proteins and DNA, decreasing protein kinase C (PKC) activity, and encouraging the release of GSH to kill cancer cells, SeNPs increase the cytotoxic action of Se compounds. The immune system of cancer patients can also be strengthened by Se, which can enhance the production of interleukin-2 (IL-2), lymphocyte lymph cytokines, interferon, and cytokines, as well as the body's ability to produce IgG, IgM, and other antibodies that can destroy cancer cells.

**Fig. 7:**
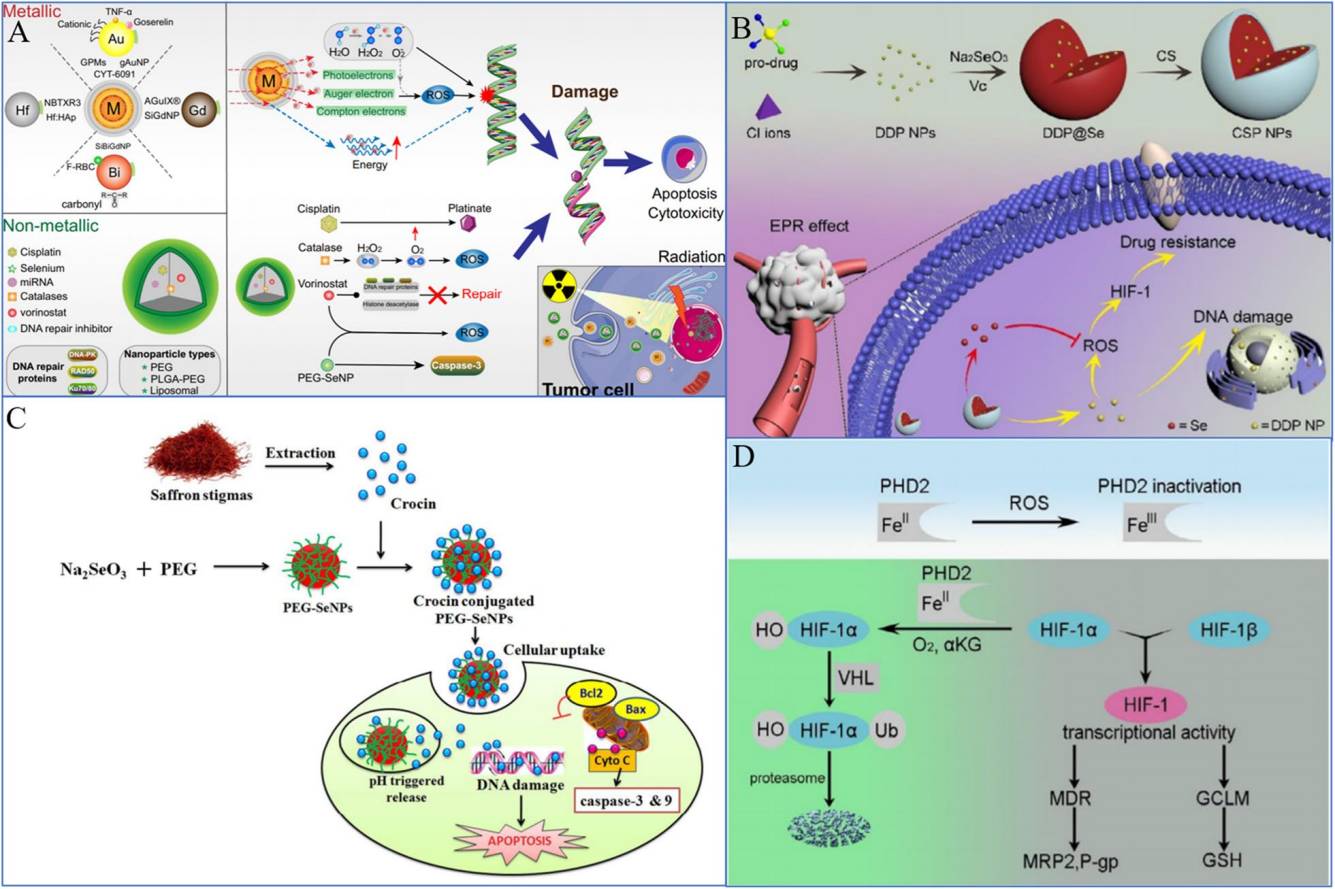
Direct cancer cell-killing activity of SeNPs. **A** Nonmetallic NPs encapsulated in combination with RT further induce DNA damage, prevent rapid DNA repair, and lead to more apoptosis [191]. Copyright 2020, The Author(s). **C** PEG-SeNPs induce apoptosis through mitochondria-mediated pathways [111]. Copyright 2019, The Royal Society of Chemistry. **B**, **D** ROS-dependent regulation of HIF-1 activity by PHD2 (chitosan-coated Se/DDP nanoparticles [CSP NPs]) [112]. Copyright 2019, Elsevier B.V

At present, Se nanomedicine contains the following drugs: DOX, 5-FU, irinotecan, cisplatin and paclitaxel [191–193]. Combining SeNPs and irinotecan, Gao et al. demonstrated greater cytotoxicity against HCT-8 tumor cells, significantly elevated p53 expression levels, and increased DNA sensitivity of HCT-8 cells to cause apoptosis [106]. The surface modification of 5-FU can dramatically increase the uptake of SeNPs by endocytosis according to studies by Liu et al. [104]. Strong selectivity and efficient growth inhibition of cancer cells are both characteristics of 5-FU@SeNPs. The mitochondria-mediated route to induce apoptosis can also cause caspase-dependent and ROS-dependent apoptosis of A375 cells. SeNPs and DOX therapy were used in a study by Xia et al. to increase the cytotoxicity of A549 cells and specifically target tumor cells to cause apoptosis [192]. To demonstrate how the release rate of paclitaxel was accelerated and how this improved the ability of A549 cells to absorb the drug and, simultaneously, reduce its toxicity [194], Zou et al. combined SeNPs with paclitaxel [195]. A549 cell apoptosis can be induced by HA-Se@PTX, which also suppresses A549 cell proliferation, migration, and invasion. The same result is also seen with cisplatin [191]. SeNPs@LNT also generates a positive therapeutic impact on lung adenocarcinoma [18] and is the focus of our research (Fig. 7).

### Synergistic effects of SeNPs and RT

**Fig. 8:**
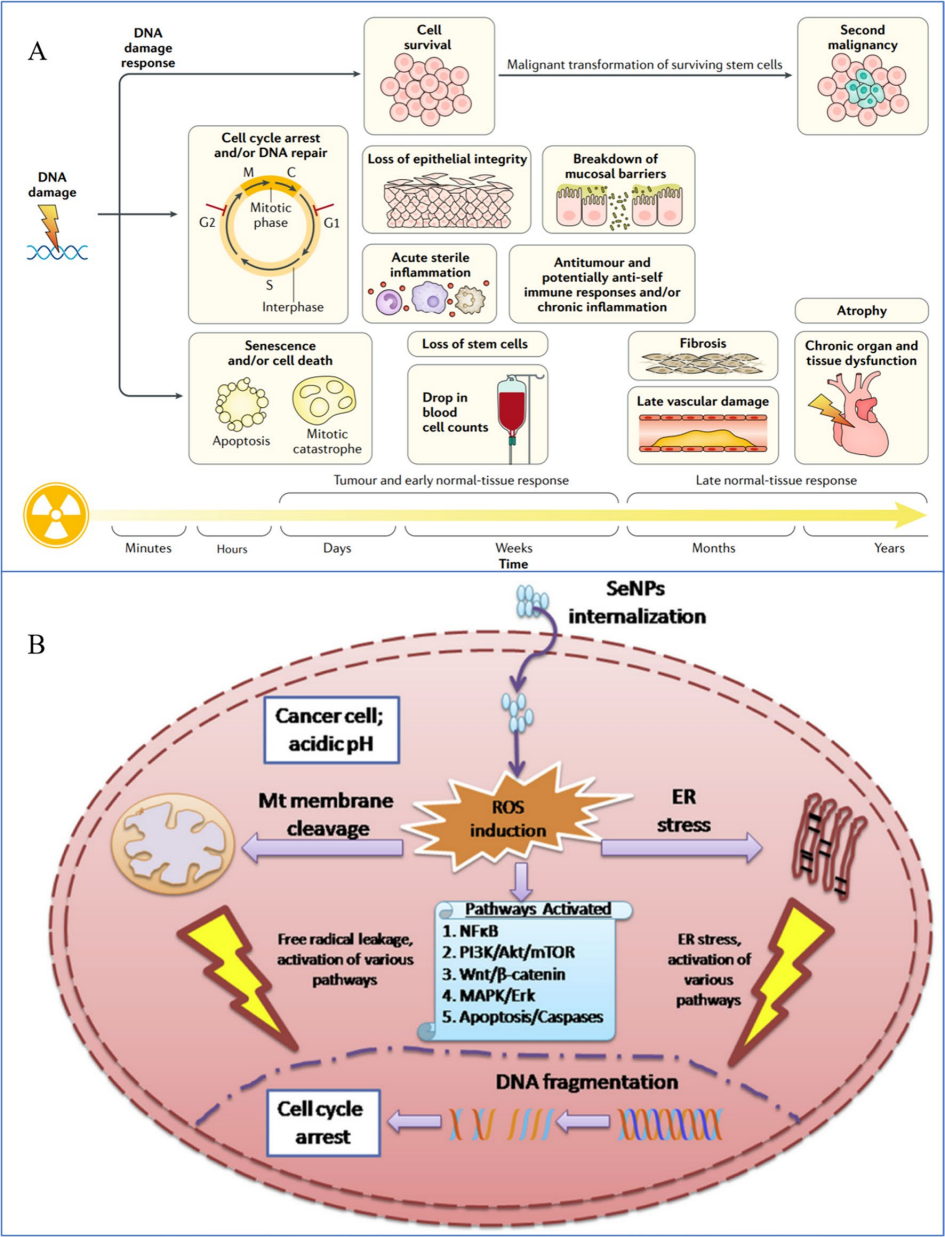
Synergistic effects of SeNPs and RT. **A** RT damaged DNA to destroy cell proliferation and gradually induced cell apoptosis over time [196]. Copyright 2019, Springer Nature Limited. **B** SeNPs kill cells by ROS in conjunction with RT [193]. Copyright 2019, Elsevier Masson SAS

As a proven treatment option for lung cancer, both palliative and curative RT is frequently used in conjunction with other therapies, including surgery, chemotherapy, or immunotherapy [197, 198]. In most cases, RT is combined with surgery and radiation, including preoperative radiation to shrink the tumor, preoperative radiation to facilitate surgical resection, intraoperative radiation to precisely deliver large doses of ionizing radiation (IR) to the tumor site while minimizing adverse effects on normal tissues, and postoperative radiation to decrease recurrence risk. [199–203]. In most cases, RT is a form of therapy that employs IR, which is typically used to describe high-energy photon radiation, such as X-rays and gamma (g) rays, as well as particle radiation such as alpha or beta particles, carbon ions, electron, proton, or neutron beams [204–206]. IR has the ability to disrupt biomolecules directly, including proteins, lipids, and DNA. This can stop cell division and proliferation as well as cause necrosis or apoptosis in some cells. In the meantime, the byproduct of radiolysis, ROS, can also damage biomolecules via free radicals. To target and eliminate tumor tissues, any of these radiation options can be used. This approach may be effective in reducing and eliminating cancerous cells, but it may also damage normal tissues adjacent to the site, which may result in toxic effects. Complications of RT include fatigue, anorexia, bone marrow injury, shortness of breath, cough and dyspnea. Furthermore, the degree of radiation damage to tissue cells is directly related to the rate of cell proliferation, the oxygen supply to the tissue, and the dose of irradiation. The higher the cell proliferation, the greater the oxygen supply of tissue cells, and the greater the sensitivity to radiotherapy. As a solid tumor, lung cancer contains 10% to 50% anoxic cells that are resistant to radiation, which makes RT alone ineffective at eradicating tumor cells and risks recurrence. Therefore, more precise RT techniques have emerged, such as intensity-modulated RT, which may decrease toxicity, but some patients still experience adverse reactions (e.g., bone marrow suppression, nausea, and vomiting hindrance) [196, 207, 208]. The use of RT alone to cure tumors is, however, difficult due to a variety of obstacles, such as cancer stem cells, tumor heterogeneity, angiogenesis and vasculogenesis, metabolic alterations, and complications [209–211]. A way to overcome these obstacles is to introduce radiosensitizers, which are molecules or materials that can enhance the radiosensitivity of tumor cells. With the development of nanotechnology, nanomaterials possessing good radiosensitizing effects and metabolic properties are being developed. SeNPs have attracted increasing attention in the past decade due to their high bioavailability, low toxicity and novel therapeutic properties (Fig. 8).

It has been reported that the effect of SeNPs combined with RT on the proliferation of non-small cell lung cancer (NSCLC) cells was greater than that of SeNPs exposure treatment alone or irradiation alone, suggesting that SeNPs and RT exhibit a synergistic effect in inhibiting cell proliferation activity or promoting each other [46]. Furthermore, the combination of the two could also exert other anticancer activities, including inhibition of invasion and migration and promotion of apoptosis in NSCLC cells.

Therefore, we fabricated X-ray-responsive SeNPs with significant radiosensitization effects by taking advantage of PEG as a surface decorator and template. In addition, studies have been carried out to examine the application potential of PEG-SeNPs as radiosensitizers. We found that X-ray irradiation (8 Gy) or PEG-SeNPs (10 μM) alone induced only slight growth inhibition on HeLa cells, in which the cell viability was 79%, 71%, and 54% in cells treated with 20, 40 and 80 μM PEG-SeNPs, respectively. In contrast, cotreatment of the cells with PEG-SeNPs and X-ray irradiation significantly enhanced the inhibition of cell growth at the same concentration of PEG-SeNPs. In particular, cotreatment with 20 μM PEG-SeNPs and X-rays significantly suppressed cell viability to 39%. The results of microscopic examination of cells also demonstrated consistent morphological changes after combined treatment with PEG-SeNPs and X-rays, such as cell shrinkage, rounding, and the appearance of apoptotic bodies. Moreover, 20 μΜ PEG-SeNPs in combination with X-rays induced an obvious increase in the sub-G1 apoptotic fraction (41.6%) and G2/M phase arrest (21.1%) [107].

On the other hand, we further found that the nanosystem could significantly induce intracellular ROS generation in a time-dependent manner, increase oxidative stress levels, and directly bind to DNA, leading to an effective radiosensitizing effect. In lung cancer, it has also been confirmed that 20 μM PEG-SeNPs combined with X-rays generates effective anticancer effects [212]. Collectively, 20 μM PEG-SeNPs exhibited significant radiosensitization when irradiated with X-rays and synergistically enhanced the antitumor effects of radiation by inducing cell apoptosis and arresting cell cycle progression. Furthermore, we found that angiogenesis plays an important role in the growth, invasion, and metastasis of pulmonary tumors, which results in the majority of lung cancer deaths. Thus, antiangiogenesis therapy may be an effective strategy for regulating pulmonary tumor growth and metastasis [213, 214]. Bevacizumab (Avastin™, Av), as a humanized monoclonal antibody, can inhibit angiogenesis by accurately targeting vascular endothelial growth factor (VEGF), which can be used as tumor starvation (antiangiogenesis) therapy to further regulate the formation and growth of new blood vessels in and around tumor tissue [215]. The rationale for AV inhibition of tumor growth is as follows: first, it binds to VEGF secreted by angiogenic tumors; second, it inhibits AV binding to VEGF receptors in vascular endothelial cells; and finally, it inhibits VEGF-induced cell proliferation, survival, permeability, nitric oxide as well as migration and tissue factor production [216]. Therefore, to further improve the radiosensitization properties and antitumor effects of SeNPs, we rationally designed and synthesized RBCs@Se/Av that combined PEG-SeNPs and Av antibody encapsulated within RBC membrane vesicles to simultaneously enhance the efficiency of cancer RT and antiangiogenesis. In response to X-rays, the nanosystems passively accumulated within cancer cells and were then activated. In line with expectations, treatment with RBCs@Se/Av and X-ray resulted in the production of ROS and the activation of the p53 pathway, which resulted in apoptosis of cancer cells. Furthermore, RBCs@Se/Av with X-ray irradiation not only do not cause obvious histological damage to major organs but also show centrally effective anticancer efficacy [113].

This method can effectively solve systemic side effects and improve the efficiency of treatment. In addition, the study not only focuses on the treatment rate but also covers the basics of the treatment mechanism. However, the reduction in the long-term recurrence rate remains to be further studied.

### Synergistic effects of SeNPs and chemotherapy

Chemotherapy is the term used to describe the administration of chemical medications to treat lung cancer. Chemotherapy is a type of systemic treatment because once anticancer medications enter the body, they are promptly dispersed throughout the body to destroy both nearby local tumors and far-off metastatic tumors. The preferred clinical therapy for NSCLC is chemotherapy coupled with radiation or immunotherapy. Chemotherapy is the main therapeutic choice for SCLC. Consequently, chemotherapy is crucial to the complete treatment of lung cancer. Antineoplastic drugs, such as DOX, paclitaxel (PTX), platinum analogs, gemcitabine, and pemetrexed, have been used widely in the treatment of lung cancer as first-line antineoplastic drugs with good results. However, most chemotherapeutic drugs are highly toxic and have side effects, which make it difficult for patients to tolerate or continue treatment completion. Furthermore, it is known that primary and acquired drug resistance in cancer cells can lead to tumor recurrence and metastasis, thus limiting the anticancer properties of chemotherapeutics [219–222]. Therefore, the chemotherapies currently available have the following primary limitations: cancer cells develop adaptive chemoresistance over time, and normal cells are subject to nonspecific toxicity [223].

Chemosensitization is the strategy used widely to enhance the activity of one drug by combining it with another drug to overcome chemoresistance. Chemotherapeutic sensitizers should be less toxic, multitargeted, and able to sensitize cancer cells to chemotherapeutic drugs by inhibiting one or more signaling pathways involved in chemoresistance, preferably multiple pathways. Therefore, the search for chemotherapeutic sensitizers has important scientific significance and application value. Numerous investigations have shown that both the tissue and cell distribution profiles of anticancer drugs can be improved by nanotechnology [224]. Nanosized anticancer drugs displayed increased antitumor efficiency and reduced serious side effects [225]. Recently, Zhou et al. synthesized SeNPs with hyaluronic acid (HA) to prepare tumor-targeted delivery vehicle HA-SeNPs and loaded PTX in HA-SeNPs to fabricate functionalized SeNPs HA-SeNPs@PTX, which showed excellent chemosensitizing activity and low toxicity [195]. Since the HA receptor CD44 expressed by A549 cells is significantly higher than that in normal human cells, HA-SeNPs@PTX has superior guided selectivity for A549 cells. HA-SeNPs@PTX can significantly improve the anticancer effect of PTX in NSCLC. The calculation of the comprehensive index value showed that HA-SeNPs@PTX enhanced the inhibition of PTX on A549 cell growth. The synergistic effect of the two is related to more significantly inducing caspase-mediated apoptosis, blocking the G2/M cell cycle and inhibiting cell proliferation. PTX is released faster under acidic conditions. This may be due to the increased protonation of HA-SeNPs under acidic conditions, which weakened the electrostatic attraction between PTX and HA-SeNPs; thus, acidic conditions facilitated the release of PTX from HA-SeNPs. Such acid-dependent drug release features of HA-SeNPs@PTX are quite favorable for cancer treatment. In addition, HA-SeNPs and HA-SeNPs@PTX showed low cytotoxicity and great biocompatibility, respectively. The results indicated that HA-Se@PTX exhibits significant potential for lung carcinoma treatment. Similarly, Xia et al. designed SeNPs that were modified with cyclic peptide (Arg–Gly–Asp–D-Phe–Cys [RGDfC]) to fabricate tumor-targeting delivery carrier RGDfC-SeNPs [192]. DOX is a very common anticancer drug used clinically [226, 227]. Nevertheless, the anticancer efficacy of DOX is not as ideal as expected, partly because of its lack of targeted specificity, poor solubility, inadequate drug accumulation in the tumor, and serious side effects. DOX was loaded onto the surface of RGDfC-SeNPs to improve the antitumor efficacy of DOX in NSCLC therapy. The anticancer mechanism of this nanosystem is similar to that of HA-SeNPs@PTX, both of which are related to caspase-mediated apoptosis and G2/M cell cycle arrest [192].

The main regulation of the cell cycle and apoptosis is ROS-mediated DNA damage, and p53 and MAPK are the main pathways of ROS-mediated DNA damage-induced apoptosis [228]. SeNPs can upregulate p53 and regulate the MAPK pathway.

In addition to its role as a transcription factor, p53 is involved in the regulation of a number of genes associated with cell cycle arrest and apoptosis [229]. The acetylation and phosphorylation of p53 play an important role in the regulation of apoptosis. For instance, phosphorylation of p53 at Ser 15 can lead to cell apoptosis caused by chemotherapeutic drugs and chemopreventive agents, especially seleno-compounds [230]. Huang et al. synthesized Tf-conjugated SeNPs and showed that they could be used as a cancer-targeted drug delivery system to achieve enhanced cellular uptake and anticancer efficacy. As a targeting ligand, Tf significantly enhances the cellular uptake of DOX-loaded SeNPs through clathrin-mediated and caveolae/lipid raft-mediated endocytosis in cancer cells overexpressing transferrin receptors and increases their selectivity between cancer and normal cells. DOX-loaded and Tf-SeNPs exhibit unprecedented, enhanced cytotoxicity toward cancer cells through the induction of apoptosis with the involvement of intrinsic and extrinsic pathways. Internalized and externalized Tf-SeNPs significantly upregulated the phosphorylation of p53 at the Ser15 site and triggered the phosphorylation of histones at the Ser139 site, indicating that Tf-SeNPs trigger cancer cell apoptosis through DNA damage-mediated p53 activation. In addition, internalized Tf-SeNPs suppressed the expression of Bcl-xl, a prosurvival member of the Bcl-2 family of proteins. Bcl-2 family proteins can regulate outer mitochondrial membrane permeability and control the on/off intrinsic apoptotic pathway. Therefore, Tf-SeNPs induce mitochondrial dysfunction, which leads to the overproduction of ROS and activates mitochondrial-mediated apoptosis. With further research, Huang et al. also found that MAPK pathways may also play an important role in Tf-SeNP-induced apoptosis [47].

**Fig. 9:**
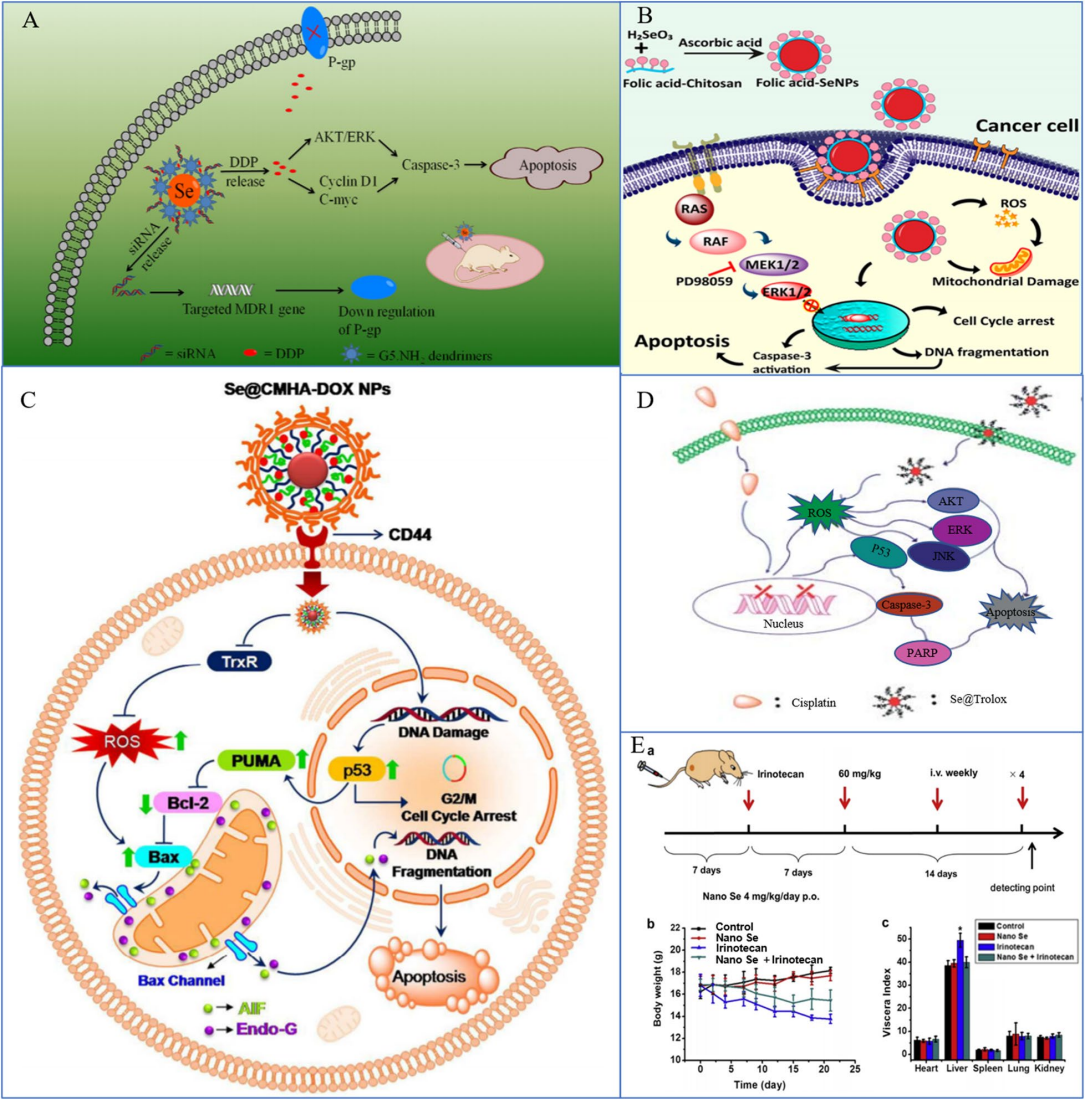
Synergistic effects of SeNPs and chemotherapy. **A** SeNPs double deliver MDR1 siRNA and DDP to reverse drug resistance [217]. Copyright 2014, Acta Materialia Inc. **B** FA@SeNPs and a MAPK-pathway inhibitor for dual targeting of cancer cells [114]. Copyright 2019, American Chemical Society. **C** Schematic diagram of Se@CMHA-DOX NP-induced apoptosis in cancer cells [218]. Copyright 2017, American Chemical Society. **D** Se@Trolox blocks cisplatin-induced signaling pathways [124]. Copyright 2013, Royal Society of Chemistry. **E** SeNPs combined with irinotecan can reduce irinotecan toxicity and treat mice [106]. Copyright 2014, Elsevier Ltd

MAPK is a serine/threonine protein kinase that plays a regulatory role in many cell activities, such as growth and proliferation, cell differentiation, cell movement and death [231]. Moreover, as noted earlier, MAPK is among the main pathways for ROS-mediated DNA damage to induce cell apoptosis [228]. The MAPK pathway mainly includes the following branch routes: ERK, JNK, p38 and AKT. Tf-SeNPs exhibited differential effects on the phosphorylation of p38, JNK, ERK, and AKT. Phosphorylation of the proapoptotic kinase p38 displayed a trend toward upregulation in a dose-dependent manner. In contrast, phosphorylation of the antiapoptotic ERK was effectively suppressed by Tf-SeNPs, while the phosphorylation of JNK and AKT was not affected by Tf-SeNPs. Taken together, the results indicated that internalized Tf-SeNPs trigger intracellular ROS overproduction, thus activating the p53 and MAPK pathways to promote cell apoptosis [47] (Fig. 9).

In addition, SeNPs combined with chemotherapy can antagonize the side effects of chemotherapy. Cisplatin is a highly effective chemotherapeutic agent used widely in the treatment of lung cancer. However, the most common adverse effect limiting the clinical use of cisplatin is nephrotoxicity, which develops primarily in the S3 segment of the proximal tubule and impairs the patient’s quality of life; moreover, in patients with preexisting conditions, nephrotoxicity can even be life-threatening [232, 233]. Research has indicated that cisplatin-related nephrotoxicity may be mainly caused by oxidative stress [234]. Treatment with cisplatin resulted in the generation of ROS, such as superoxide anion and hydroxyl radicals, and renal lipid peroxidation [235]. Moreover, several studies have demonstrated the protective effect of natural or synthetic antioxidants against cisplatin-induced nephrotoxicity [236, 237]. 6-Hydroxy-2,5,7,8-tetramethylchroman-2-carboxylic acid (Trolox) is a water-soluble analog of alpha-tocopherol with a chromatid ring that has been studied extensively for its protective effects against oxidative stress-related diseases. Trolox is poorly utilized due to its poor water solubility and poor stability when exposed to oxygen-containing environments due to its phenolic hydroxyl group. In recent years, due to the development of nanotechnology, the stability of Trolox can be improved effectively by nanocrystallization [238]. Chen et al. found that Trolox surface-functionalized SeNPs (Se@Trolox) could also play a similar role [124], which could effectively reduce cisplatin-induced nephrotoxicity. Through inhibition of ROS-mediated p53 phosphorylation, Se@Trolox prevented the caspase-mediated apoptosis induced by cisplatin. Intrinsically, Se@Trolox effectively protected HK-2 cells from damage by regulating the Akt and MAPK pathways. Furthermore, Chen et al. also reported a simple method for the functionalization of SeNPs by self-assembly of 11-mercapto-1-undecanol (Se@MUN) to achieve enhanced antioxidant activity and demonstrate antagonism against cisplatin-induced nephrotoxicity [115]. The mechanism for the action of Se@MUN involved significantly reducing the decreased HK-2 cell viability induced by cisplatin, including the sub-G1 peak, nuclear concentration, and DNA fragmentation. Se@MUN also effectively blocked the activation of caspase-3 in cells induced by cisplatin, and compared with SeNPs, Se@MUN showed higher free radical scavenging activity and higher cell uptake in normal human cells. Therefore, the nanocrystallization of org-Se has potential application value in preventing cisplatin-induced injury.

Therefore, SeNPs combined with chemotherapy drugs can not only enhance the efficacy of drugs but also improve the accuracy and targeting, reduce side effects, and antagonize the side effects of chemotherapy drugs, which is promising research for clinical application.

### Application of SeNPs in photodynamic and photothermal tumor therapy

Recently, research on PTT, a newly developed and encouraging therapeutic strategy, has achieved many breakthroughs in the treatment of cancer. In PTT, photothermal transduction agents (PTAs) are used to produce heat by harvesting the light's energy and converting it into heat to increase the surrounding environment's temperature and cause cancer cells to die [241, 242]. In contrast to conventional therapy modalities, PTT exhibits unique advantages in cancer therapy, including high specificity, minimal invasiveness, and precise spatial–temporal targeting [243, 244]. PTT treatment can be used to eliminate cancer cells in a primary tumor or local metastasis in nearby lymph nodes, as well as to treat cancer cells that have metastatic spread, in conjunction with current therapeutic modalities [245, 246]. More notably, PTT is a highly effective and noninvasive therapy that is capable of eliminating various types of cancers [247]. Moreover, it has been suggested that tumors became more susceptible to chemotherapy and RT after PTT, demonstrating a synergistic effect when used in combination. The possible mechanism to explain these effects is that while PTT destroys cancer cells, it also causes a certain degree of damage to stromal cell components, including tumor vasculature, inflammatory cells, stromal fibroblasts and lymphocytes. Under these conditions, residual cancer cells may not readily adapt to the new microenvironment, and there is a window for combination therapy with pH- or hypoxia-responsive drugs [210]. On the other hand, safety is another concern for PTT against cancer. For local tissues, long-term exposure to temperatures above 43 °C is dangerous and causes irreversible, severe damage to the cells [80]. Therefore, PTT needs to be conducted at a reasonable temperature and duration to minimize damage to normal tissues.

The therapeutic efficacy of PTT is significantly dependent on PTA. An ideal PTA should have higher photothermal conversion efficiency (PCE), absorption that does not overlap with the background of the tumor, and good tumor accumulation. The occurrence of a variety of PTAs has accelerated and advances are being made in PTT studies. In particular, nano PTAs that can accumulate in tumors through enhanced permeability and retention (EPR) effects and active targeting are noteworthy [30, 248, 249]. Moreover, since nano PTAs show a higher PCE than that of small molecule PTAs, the functionalities of multiple imaging and therapy can potentially be integrated into one platform for advanced applications [250]. Chen et al. developed a stable, highly uniform in size, and nontoxic nanomaterial made of a tellurium-Se (TeSe)–based lateral heterojunction, which showed favorable photothermal stability and the potential to provide a drug carrier platform for cancer treatment [116]. To prevent off-targeting adverse effects on the surrounding tissue, which is important for its clinical translation [211], Chen et al. used 808-nm light in the near-infrared region. In all ratios studied, Te:Se at 1:1 produces uniform, oval-shaped NPs that produce high light-to-heat conversions. At this optimal ratio for maximal production of thermal energy, the Te:Se heterojunctions are completely nontoxic, which further reduces the side effects of TeSe-based PTT. Regarding safety, during the irradiation course of in vivo treatment, although the temperature was above 50 °C and skin tissue damage was observed, this kind of damage was transient and was repaired within 5 days, demonstrating that TeSe-based PTT is safe. Systemic delivery of Te@SeNPs in mice showed highly specific accumulation in tumors relative to other healthy tissues. Upon exposure to light, Te@SeNPs almost completely eradicated lung cancer in preclinical models [116].

**Fig. 10:**
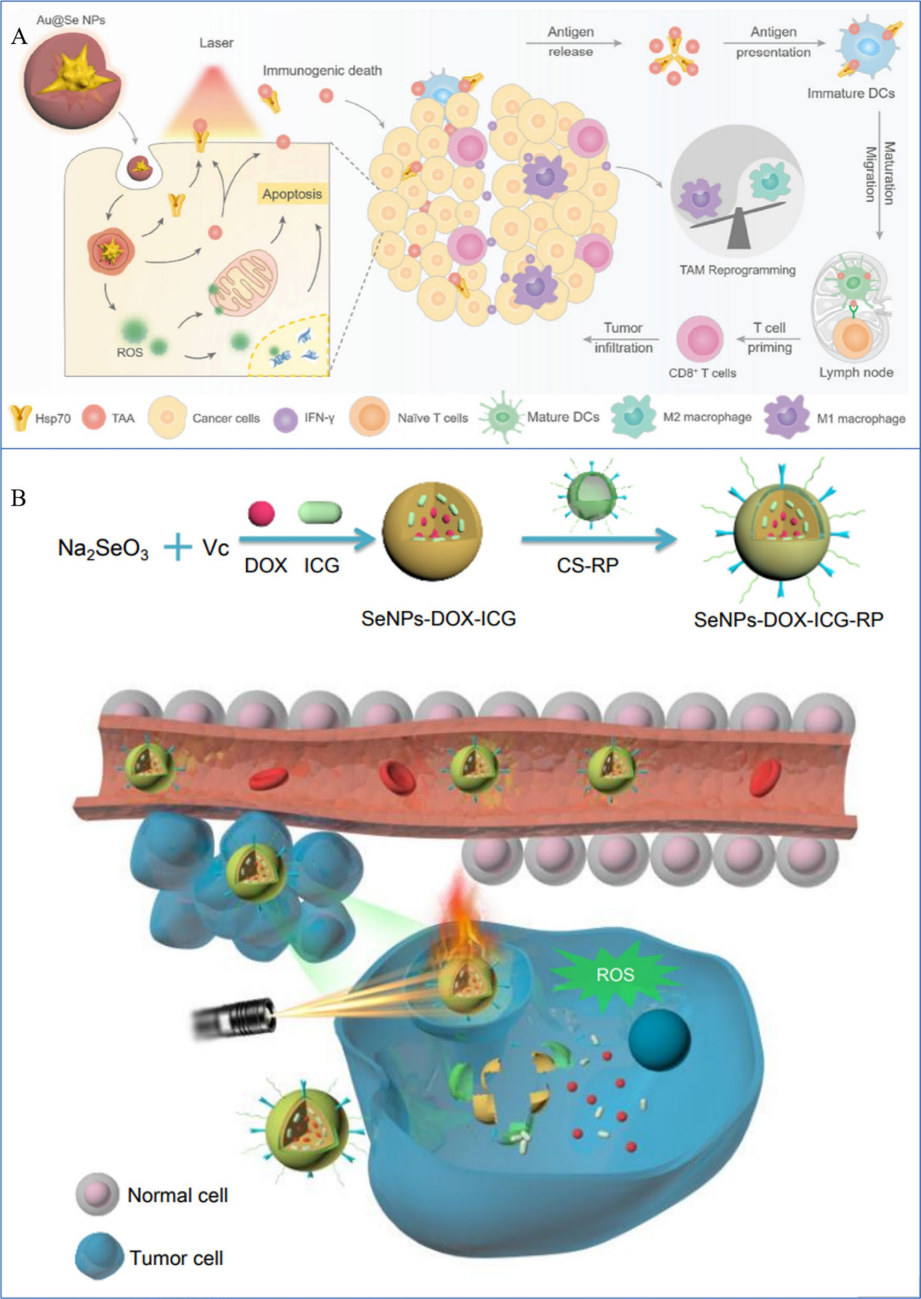
Photodynamic and photothermal tumor therapy. **A** Immunogenic nanotherapeutic agent Au@Se NPs are used for PTT-triggered immunotherapy [239]. Copyright 2020, Elsevier Ltd. **B** Mechanism of tumor targeting SeNPs by near-infrared laser irradiation [240]. Copyright 2018, Wiley‐VCH

Moreover, SeNPs can be designed as a sequentially triggered system that combines PPT with chemotherapy to achieve precise drug delivery by chemo-photothermal combination. To construct a multifunctional nanodrug delivery system (i.e., SeNPs-RP), Fang conjugated RC-12 and PG-6 to SeNPs using chitosan as the linker [240]. RC-12, which is a derivative of RGD, is a specific cancer-targeting and cell-penetrating peptide that can recognize and interact with integrin receptors that are overexpressed in various human cancer cells [251]. PG-6 (PLGALG) can be identified and cleaved by matrix metalloproteinases (MMP2 and MMP9) in the tumor microenvironment [252]. In addition, positively charged DOX molecules and negatively charged indocyanine green (ICG) molecules were loaded into the positively charged SeNPs-RP by electrostatic interactions. ICG has been reported to be harmless and an efficient therapeutic agent [253]. However, ICG, a clinical-medical diagnostic reagent that is approved by the FDA, is not an efficient PTT agent, owing to its severe photobleaching, short bloodstream circulation half-time, and low tumor accumulation rate [254]. DOX is a very common anticancer drug that is used widely in chemotherapy. Nevertheless, the anticancer efficacy of DOX is not optimal because of its lack of targeted specificity, poor solubility, inadequate drug accumulation in the tumor, and serious side effects [255]. Therefore, dual-target (RC-12 and PG-6 peptides) functionalized SeNPs loaded with both DOX and ICG would overcome the drawbacks of ICG and ensure that the chemotherapeutic drug and photothermal agent are delivered synchronously to the tumor area to produce their synergistic effect. The as-synthesized NPs exhibited good monodispersity, size stability and consistent spectral characteristics compared with ICG or DOX alone. The NPs underwent self-immolated cleavage with NIR laser irradiation and released the loaded drug due to sufficient hyperthermia. Additionally, the internalized NPs triggered intracellular ROS overproduction to induce cell apoptosis. This dual-targeted design of SeNPs loaded with both DOX and ICG might provide a feasible solution for efficient anticancer drug delivery and provide a sequentially triggered nanosystem to achieve precise drug delivery by chemo-photothermal combination (Fig. 10).

## Tumor immunotherapy based on SeNPs

### Innate immunity of SeNPs in lung cancer treatment

Se supplementation is typically immunostimulatory, as shown by a variety of metrics, such as T-cell proliferation, NK cell activity, and innate immune cell activities [259]. Although Se is essential for numerous immune cell functions, there has been no conclusive evidence that Se and selenoprotein levels influence immune system development. More thorough analysis is needed to determine whether Se influences immune responses and underlying mechanisms in lung cancer.

**Fig. 11:**
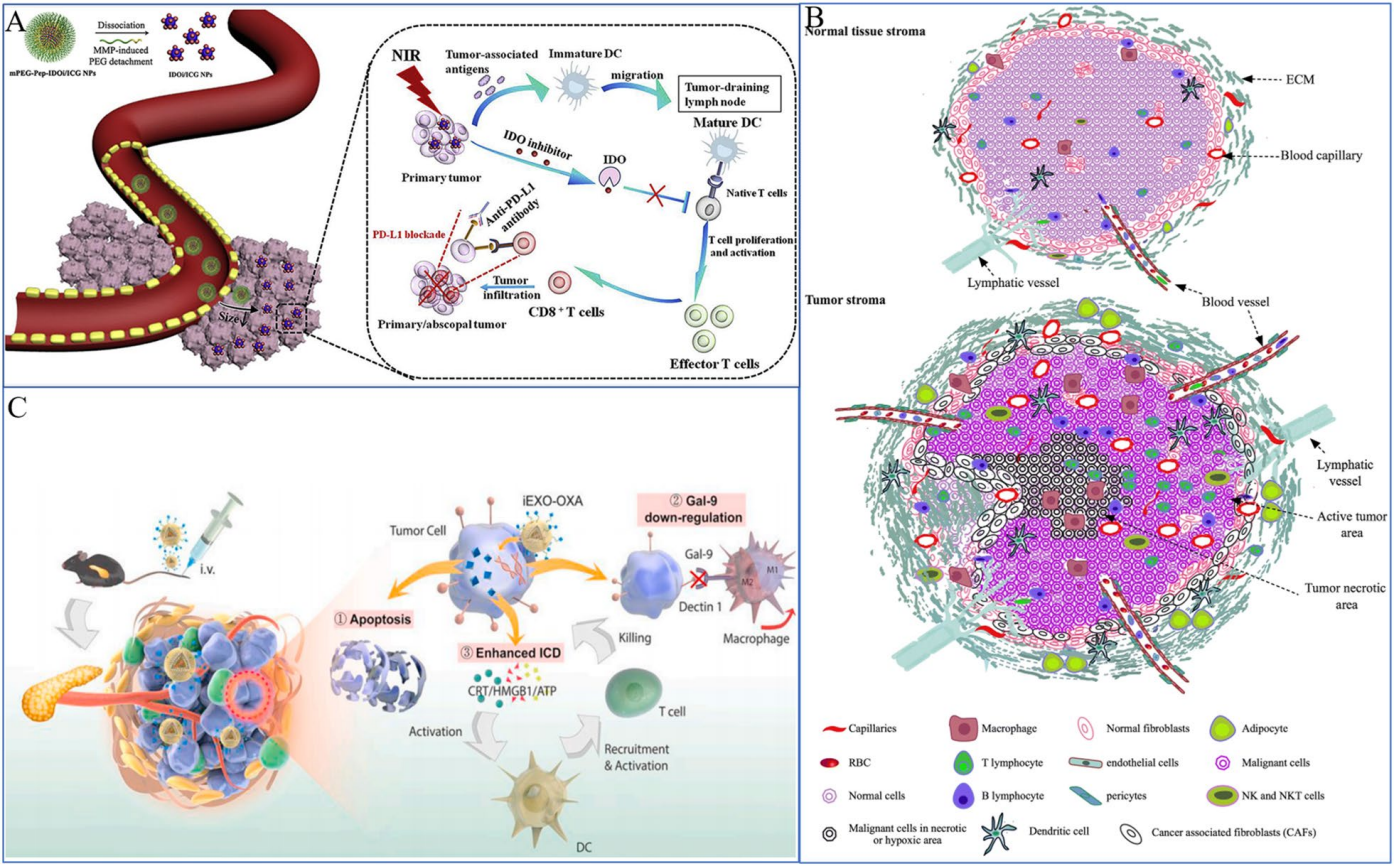
The immune microenvironment and immune cell status. The immune microenvironment is complex, including tumor cells, immune cells and the secretion of various cytokines, including a number of trace elements. **A** Prodrug nanodrugs are characterized by prolonged blood circulation time, enhanced tumor accumulation and deeper penetration. Combined with phototherapy, IDO inhibition and PD-L1 blocking, synergistic and effective antitumor immunotherapy can be achieved [256]. Copyright 2020, Elsevier Ltd. **B** Tumor microenvironments include high infiltration of immune cells, cancer cells, and CAFs/TAFs and increased deposition of ECM protein in the interstitial tissue [257]. Copyright 2018, The Author(s). **C** Exosomes enhance immunotherapy and reprogram the tumor microenvironment [258]. Copyright 2020, Elsevier Ltd

**Fig. 12:**
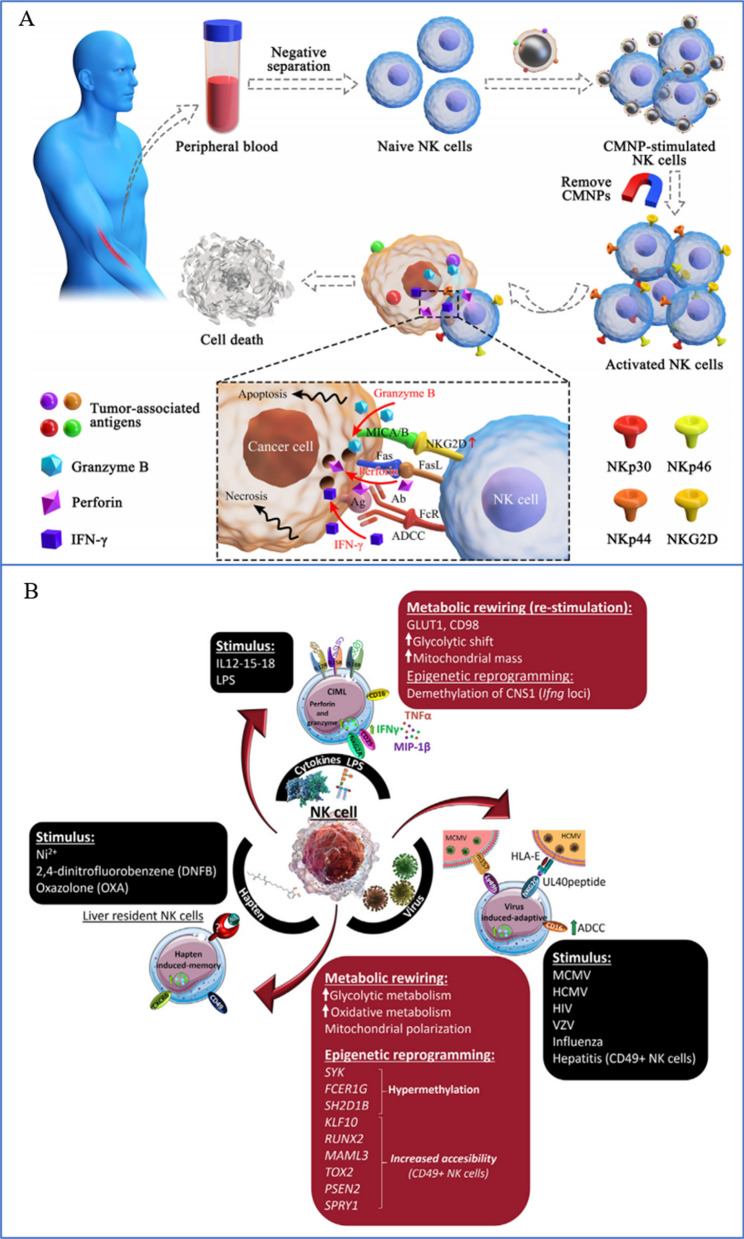
SeNPs and NPs can activate NK cell activity in vivo, release factors, and upregulate receptors to play an immunotherapeutic role. **A** Magnetic nanoparticles encapsulated in tumor cell-derived membranes are used to enhance NK cell-based immunotherapy [260]. Copyright 2020, Elsevier Inc. **B** NK cells exhibit three types of innate memory and memory-like responses depending on the initial stimulus [261]. Copyright 2021, Elsevier B.V

NK cells are key effector cells in tumor immunotherapy because they are a crucial part of the innate immune system [262]. Se and Se compounds can control the activation and antitumor activity of NK cells in the tumor microenvironment (TME) (Fig. 11). Se stops para fibrin from forming nonenzymatically around tumor cells, making the tumor more susceptible to immune surveillance [263, 264]. Additionally, it stimulates the NK cell population in the TME [265] (Fig. 12). With an improved EPR impact, SeNPs increased the targeted delivery of Se in target cells [191]. According to Gao et al. [266], SeNPs may act as immune checkpoint inhibitors with direct anticancer effects on lung metastasis as well as immunomodulatory activity. Their findings showed that in SeNPs, inhibiting HLA-E expression at the tumor site specifically results in the immunological augmentation of NK cells. In our previous study [18], we discovered that SeNPs@LNT increased the number of NK cells in MPE from lung adenocarcinoma (MPE-LA) compared to the control group (30.8% vs. 15.2%). SeNPs@LNT could be transformed to SeCys_2_ and Se(IV, ${\text{SeO}}_{3}^{2 - }$) in the immune cell lysate to regulate the secretion of cytokines by multiple immune cells (such as NK and tumor-associated macrophages [TAMs]) to reprogram the inflammatory microenvironment of MPE-LA.

TAMs, as innate immune cells, also play an important role during an encounter with lung cancer cells. TAMs are heterogeneous cells that may acquire opposite functions in response to different TME signals, and they can differentiate into TAM1 or TAM2. TAM-polarized M2 macrophages inhibit cancer cell apoptosis in the MPE microenvironment, while tumoricidal M1 macrophages exhibit preferential immune activation [267]. In a previous study, we also found that treatment with SeNPs@LNT for MPE-LA helped to re-educate M2 macrophages into an M1 phenotype (Fig. 14C).

**Fig. 13:**
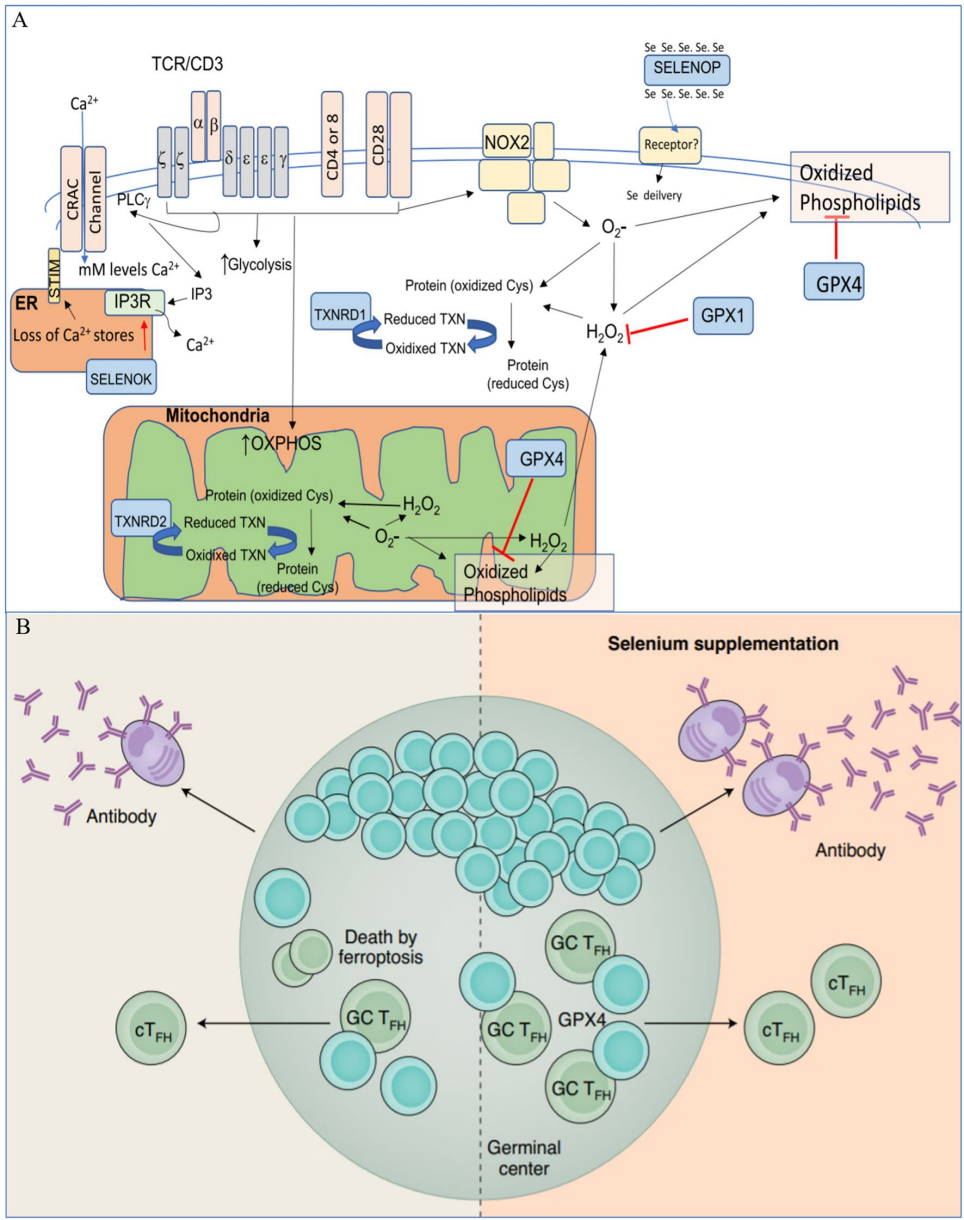
SeNPs and NPs can activate T-cell activity in vivo, release factors, and upregulate receptors to play an immunotherapeutic role. **A** The role of selenoproteins in T-cell biology [268]. Copyright 2021, Springer Nature America, Inc. **B** Selenium supplementation boosted TFH cells in mice and humans [269]. Copyright 2020, Elsevier Ltd

T lymphocytes play a central role in lung cancer immunity. They kill malignant cells through T-cell receptor (TCR) recognition of specific antigenic peptides on the surface of target cells [270]. T cells secrete cytokines, including IFN-γ, TFN-α, IL-2, IL-10, IL-4, and IL-17 [271–274]. Hu et al. reported the mechanism by which lysosomal SeNPs regulate mitochondrial metabolism and biosynthesis, and at the same time, they stabilized the microtubule structure and further enhanced the cytotoxicity of γδ T cells by upregulating tubulin-α acetylation [50]. Wang et al. [239] reported that the synergistic effect between SeNP-mediated chemotherapy and AUNSS-induced PTT could support T-cell activation and kill tumors [275] (Fig. 13).

**Fig. 14:**
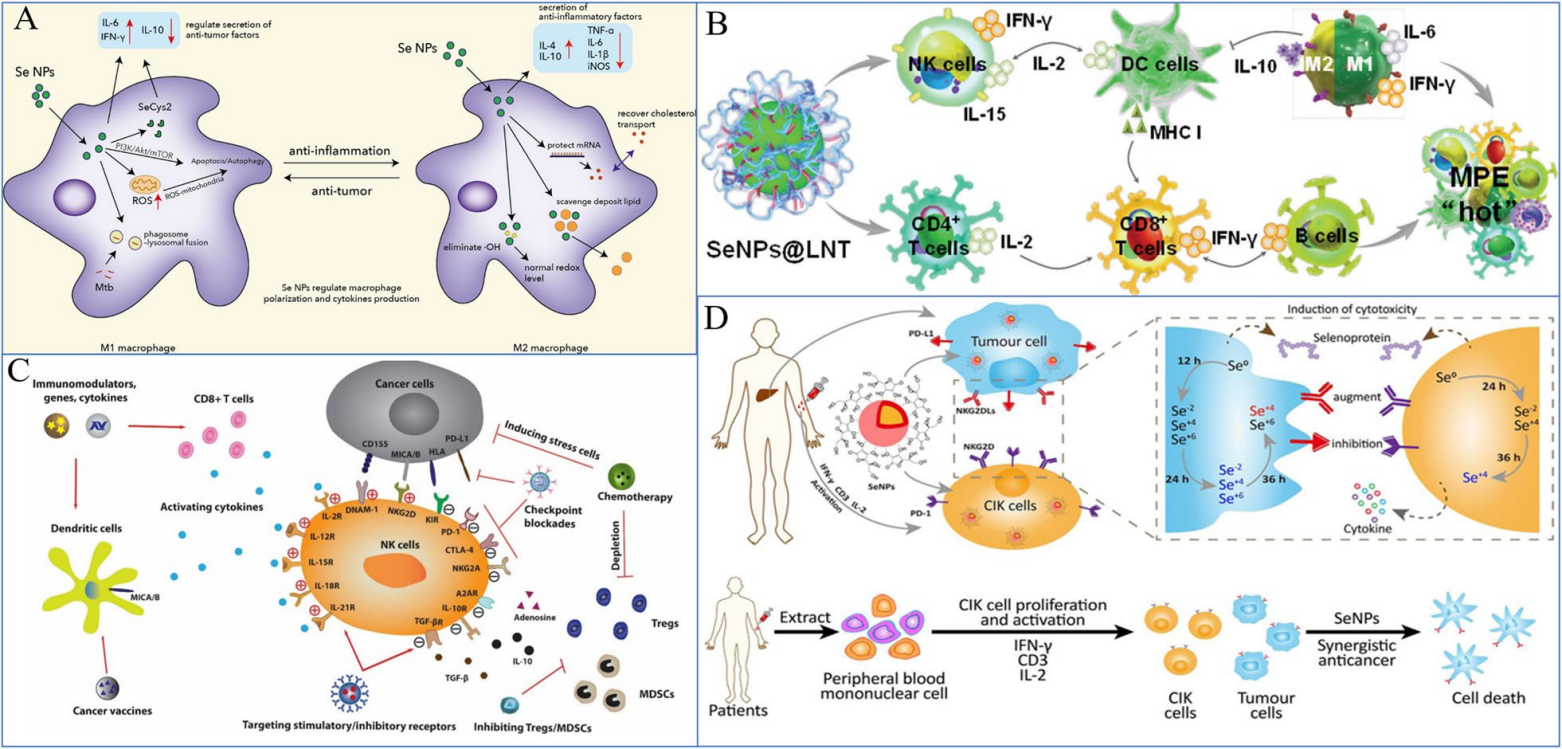
SeNPs play an immune role in coordination with other cells. **A**, **C** SeNPs and NPs enhance the activity of other cells to promote an antitumor effect [285, 286]. Copyright 2022, Chen, Yang, Fan, Jin, Liao, Li, Liu, Liang, Zhang, Xu and Pi. Copyright 2020, The Pharmaceutical Society of Korea. **B** SeNPs@LNT promote NK cell secretion of cytokines and macrophage transformation [18]. Copyright 2021, Wiley‐VCH **D** SeNPs with CIK cells for effective cancer immunotherapy [45]. Copyright 2020, Elsevier Ltd

SeNPs regulate these innate immune cells to achieve antitumor effects, and their antitumor mechanism has been reported in the literature. However, the research involves some shortcomings, including the following: the number of immune cells may not be sufficient, so research has also shown that SeNPs regulate the proliferation of immune cells, that is, adoptive cellular immunotherapy (ACI) (Fig. 14A, B).

### Application of SeNPs in ACI

ACI is a therapeutic method in which immune cells, such as NK cells and T cells, collected from tumor patients are stimulated in vitro and then transfused back into patients [276, 277]. However, the application of nanocarriers can be equivalent to in vitro amplification of immune cells, playing a therapeutic role[278]. ACI mainly includes chimeric antigen receptor (CAR) [279], cytokine-induced killer cell (CIK) [280], and tumor-infiltrating lymphocytes. However, SeNPs mainly adopt CAR and CIK [45].

ACI based on NK cells and T cells is currently an attractive approach for cancer treatment. PD-1 is a protein on T cells, and PD-1/PD-L1 is very commonly used to treat lung cancer. Therefore, NPs mainly expand T cells, secrete more PD-1, and kill tumor cells [281]. CAR-T-cell technology is limited by the inhibitory effect of the TIME and is not effective in treating solid tumors [282]. The application of nanotechnology has successfully solved these problems. NK cells, as "companion cells", can overcome the potential for serious adverse reactions in CAR-T-cell therapy for lung cancer, such as cytokine release syndrome [283].

To simultaneously deliver targeted therapy for lung cancer, Xu et al. built a therapeutic nanoplatform using a lipid aptamer nanostructure [284]. This platform can be employed in conjunction with CAR-NK immunotherapy, demonstrating that CAR-NK can benefit from the synergistic effects of nanotechnology. Additionally, SeNPs function in CAR-NK cells. For the first time, Gao et al. accomplished immunological augmentation of NK cells in the form of Se-containing NPs in metastatic lung tumor and subcutaneous tumor models by proposing a unique strategy that utilizes the ionizing radiation response features of diselenide bonds [52]. As a result, we observed the potential for using SeNPs in CAR-NK cells. In addition, Liu et al. showed a secure and successful method [45]. SeNPs can successfully increase the persistence of CIK cells in vivo in peripheral blood, enabling effective cancer immunotherapy. In addition, the combination therapy "CIK + SeNPs" can successfully encourage more NK cells to colonize malignancies (Fig. 14D).

SeNPs can play a significant role in the immunotherapy of lung cancer by working in conjunction with ACI and enhancing innate immune function in vivo. In light of the potential of ACI, more research on the use of SeNPs in lung cancer may be conducted in the future.

## Conclusions and outlook

In general, Se is an important essential trace element for the human body [287]. It can interfere with many physiological processes by regulating selenoproteins and plays an antitumor role by inhibiting protein and DNA biosynthesis and PKC activity. SeNPs can also be used as carriers to deliver drugs to tumor sites to enhance antitumor efficacy. Se also shows potent application as a theranostic for lung cancer. SeNPs can play a role in lung cancer diagnosis, including fluorescence, MR, CT and photoacoustic imaging and other diagnostic methods, as well as treatment through direct killing, radiosensitization, chemotherapeutic sensitization, photothermodynamics, and enhanced immunotherapy. In the field of cancer immunotherapy, the application of Se is also a current hot spot of scientific research because it can directly inhibit tumor cells or indirectly kill tumor cells through activation of NK cells, T cells and other immune cells. However, importantly, although there are a large number of studies utilizing SeNPs in the diagnosis and treatment of lung cancer, most of them remain at the basic research stage, with only a few clinical trials. There are still many problems and challenges that must be faced from basic research to clinical application. In the future, it is necessary to further clarify the mechanism and strengthen the basic-to-clinical transformation.

### Problems overcome by the application of SeNPs in the diagnosis and treatment of lung cancer

Currently, increasing numbers of nanodrugs have been developed and used in the clinical treatment of lung cancer, including polymeric micelle paclitaxel [288], liposomal cisplatin [289], CRLX–101 [290], BIND–014 [291], tecemotide [292], and other drugs that are currently in clinical trials. However, Se nanomedicine is still rarely used in the clinic, which could be due to several factors. First, we still need to find more efficient, low-toxicity, simple-structure, high stability, and high bioefficiency Se nanomaterials [293, 294]. Second, there are difficulties with technological breakthroughs from the laboratory to industrial production. Obstacles to the clinical development of nanoparticles include chemistry, manufacturing and control (CMC), good manufacturing practices (GMP), and various aspects of the process from the laboratory to clinical use and commercialization. The transition from the laboratory to the clinic is often accompanied by parameter optimization and even methodological changes, so it is important to carefully consider particle size in the early design phase of nanoparticles [295]. Third, the maturity of Se nanomedicine for oncology is another challenge. These kinds of studies must be elaborated from the aspects of tumor enrichment efficiency, tumor penetration, and how to achieve controlled release of drugs. The clinical application of Se nanomedicine in lung cancer requires more effective SeNPs to be designed based on clinical needs, achieving industrial production, and effectively solving the difficulties faced with lung cancer treatments, thus defining the direction for future development.

### Future direction and challenge for Se nanomedicine in lung cancer treatment

#### Discovery of more definite and specific targets for lung cancer treatment

In recent decades, targeted therapy has resulted in significant progress in the clinical treatment of lung cancer patients with genetic mutations. However, TKIs are associated with notable side effects in some patients, and drug resistance is inevitable. In recent years, cancer immunotherapy has been among the most important advances in the field of cancer therapy. It has achieved obvious and lasting curative effects in the treatment of some patients with advanced lung cancer, but the overall efficacy rate remains to be improved. Therefore, further development of targeted drugs and immune checkpoint inhibitors is needed. Combined with nanomaterials, it is expected that more specific targets for the treatment of lung cancer will be discovered. Due to the high EPR effect of nanomaterials on solid tumors, lung cancer tissues can be targeted passively to improve drug delivery and accumulation [296, 297]. In addition, another major advantage of nanomaterials is their surface modification possibility, which contains as amino, sulfhydryl, carboxyl and other active groups on the surface and thus can be used to covalently connect with ligands and targeted receptor molecules to achieve active targeting of tumors and further improve the delivery efficiency of nanomaterials [298]. By taking advantage of these two characteristics, we can further improve the targeting effect of existing TKI drugs and immune checkpoint inhibitors on lung cancer, reduce side effects, and hopefully find more specific therapeutic targets for lung cancer.

#### Designing high-specificity Se-containing drugs

SeNPs can be used as drug carriers to deliver anticancer drugs directly to tumor sites, thus reducing toxic side effects. To date, SeNPs have been employed to carry cisplatin, DOX, paclitaxel and other drugs directly to lung cancer sites. In addition, SeNPs themselves can activate NK cells [262], T cells [270], macrophages [18] and other cells to play an immune synergistic therapeutic role. In addition, SeNPs can regulate p53 [228], MAPKs [231] and other genes to further activate ROS-related pathways and enhance the therapeutic effects. However, the specific mechanisms by which immune cells are used as therapeutic targets are not well understood, nor are the receptors on immune cells. Most importantly, these studies are limited to preclinical studies and have not yet been developed in the clinical setting. Therefore, more specific Se-containing drugs are needed to treat lung cancer, either by directly acting on tumor sites and specific genetic targets of lung cancer cells or by acting on receptors on immune cells.

#### Developing new treatment strategies through multimodal integration therapy

At present, nanomedicine-based treatment of lung cancer has been integrated with chemotherapy, chemo-sensitization, photodynamic therapy, immunotherapy and other treatment methods, all of which have shown great potential for lung cancer treatment. The use of immune-synergistic therapy may become the focus of future research to develop new receptor/ligand targets, new immune cell therapy models, and more new and effective therapeutic strategies through combination with physical therapy, traditional Chinese medicine, and surgery to achieve better treatment efficacy.

## Data Availability

Not applicable.

## Abbreviations

Se: Selenium
SCLC: Small cell lung cancer
EGFR: Epidermal growth factor receptor
TIME: The tumor immune microenvironment
Inorg-Se: Inorganic selenium
Org-Se: Organic selenium
Doxil: Doxorubicin hydrochloride liposome nanomedicine
SeNPs: Selenium nanoparticles
COPD: Chronic obstructive pulmonary disease
ARDS: Acute respiratory distress syndrome
H_2_Se: Hydrogen selenide
CH_3_SeH: Methylselenol
MSA: Methylseleninic acid
SOD: Superoxide dismutase
NF-kB: Nuclear factor kappa beta
GPX: Glutathione peroxidase
NPs: Nanoparticles
PLAL: Pulsed laser ablation in liquids
Na_2_SO_3_: Sodium sulfite
Blg: β Lactoglobulin
CS: Chitosan
SPS: Spiral algae polysaccharide
PSP: Polysaccharide-protein complex
SFS: Sodium formaldehyde sulfonate
EPS: Cordyceps exopolysaccharides
MPE: Malignant pleural effusion
SeO_2_: Se dioxide
Se/Ti (I): (II) and (III), Se dioxide/titanium dioxide nanocomposites
2D: Two-dimensional
TMDCs: Transition metal dichalcogenides
MoSe_2_: Molybdenum selenide
MoSe_2_(Gd^3+^): Gd^3+^-doped MoSe_2_
FRAP: Fluid on the iron-reducing antioxidant capacity
CRP: C-reactive protein
PEG: Polyethylene glycol
PAI: Photoacoustic imaging
t-Se: Trigonal Se
FRI: Fluorescence imaging
MRI: MR imaging
FeSe_2_: Iron diselenide
FDA: Food and Drug Administration
IL-2: Interleukin-2
ROS: Reactive oxygen species
RT: Radiotherapy
PTX: Doxorubicin, paclitaxel
HA: Hyaluronic acid
Tf: Transferrin
Se@Trolox: Trolox surface functionalized SeNPs
DOX: Doxorubicin
PTT: Photothermal therapy
EPR: Enhanced permeability and retention
PTAs: Photothermal transduction agents
TeSe: Tellurium-Se
PCE: Photothermal conversion efficiency
ICG: Indocyanine green
MPE-LA: MPE from lung adenocarcinoma
MAPK: Mitogen-activated protein kinase
Se@MUN: SeNPs by self-assembly of 11-mercapto-1-undecanol
RGDfC: Arg–Gly–Asp–D-Phe–Cys
TAMs: Tumor-associated macrophages
ACI: Adoptive cellular immunotherapy
TME: The tumor microenvironment
TCR: T-cell receptor
CIK: Cytokine-induced killer cell
CAR: Chimeric antigen receptor
